# Thermoneutral housing promotes hepatic steatosis in standard diet-fed C57BL/6N mice, with a less pronounced effect on NAFLD progression upon high-fat feeding

**DOI:** 10.3389/fendo.2023.1205703

**Published:** 2023-07-12

**Authors:** Olga Horakova, Gabriella Sistilli, Veronika Kalendova, Kristina Bardova, Marko Mitrovic, Tomas Cajka, Ilaria Irodenko, Petra Janovska, Karoline Lackner, Jan Kopecky, Martin Rossmeisl

**Affiliations:** ^1^ Laboratory of Adipose Tissue Biology, Institute of Physiology of the Czech Academy of Sciences, Prague, Czechia; ^2^ Faculty of Science, Charles University, Prague, Czechia; ^3^ First Faculty of Medicine, Charles University, Prague, Czechia; ^4^ Laboratory of Translational Metabolism, Institute of Physiology of the Czech Academy of Sciences, Prague, Czechia; ^5^ Institute of Pathology, Medical University of Graz, Graz, Austria

**Keywords:** non-alcoholic fatty liver disease, liver steatosis, NASH, C57BL/6N mice, obesity, thermoneutrality, metabolomics, *de novo* lipogenesis

## Abstract

**Introduction:**

Non-alcoholic fatty liver disease (NAFLD) can progress to more severe stages, such as steatohepatitis and fibrosis. Thermoneutral housing together with high-fat diet promoted NAFLD progression in C57BL/6J mice. Due to possible differences in steatohepatitis development between different C57BL/6 substrains, we examined how thermoneutrality affects NAFLD progression in C57BL/6N mice.

**Methods:**

Male mice were fed standard or high-fat diet for 24 weeks and housed under standard (22°C) or thermoneutral (30°C) conditions.

**Results:**

High-fat feeding promoted weight gain and hepatic steatosis, but the effect of thermoneutral environment was not evident. Liver expression of inflammatory markers was increased, with a modest and inconsistent effect of thermoneutral housing; however, histological scores of inflammation and fibrosis were generally low (<1.0), regardless of ambient temperature. In standard diet-fed mice, thermoneutrality increased weight gain, adiposity, and hepatic steatosis, accompanied by elevated *de novo* lipogenesis and changes in liver metabolome characterized by complex decreases in phospholipids and metabolites involved in urea cycle and oxidative stress defense.

**Conclusion:**

Thermoneutrality appears to promote NAFLD-associated phenotypes depending on the C57BL/6 substrain and/or the amount of dietary fat.

## 1 Introduction

Non-alcoholic fatty liver disease (NAFLD) represents a spectrum of disorders ranging from simple steatosis to non-alcoholic steatohepatitis (NASH), characterized by hepatocellular inflammation and ballooning, which can develop further into fibrosis, cirrhosis and/or carcinoma. It is currently the most common liver disease worldwide, with an estimated prevalence of 25% (1). While there is no approved medication for the treatment of NAFLD, the disease is closely associated with obesity and is considered to be the hepatic manifestation of metabolic syndrome (2). However, given its link to metabolic dysfunction and heterogeneous pathogenesis, it has recently been suggested that a more appropriate term might be metabolic (dysfunction)-associated fatty liver disease (3).

Preclinical rodent models are essential for understanding the pathogenic mechanisms of NAFLD development and progression as well as for testing new potential therapies. Different strategies have been used to induce NAFLD in mice, often based on the use of genetic models (e.g. ob/ob or db/db mice) or special diets, such as methionine- and choline-deficient diet, high-fat diet (HFD), high-cholesterol and cholate diet, or high-fructose diet (4–6). In addition to the fact that not all of these experimental approaches are associated with an obese phenotype, insulin resistance and/or NASH development, the degree of NAFLD severity also depends on the genetic background of the mouse strain chosen (7). Thus, the ideal mouse model for human-like progressive NAFLD combines both genetic and environmental cues. For example, recently developed mouse models of obesity-associated NASH, such as DIAMOND (8) and FATZO (now MS-NASH (9, 10)) mice, are based on stable isogenic crosses between the inbred mouse substrain C57BL/6J (B6/J) and either the 129S1/SvImJ strain (i.e. DIAMOND mice) or the AKR/J strain (i.e. MS-NASH mice), in which dietary challenges trigger the progression of NAFLD. Although inbred B6/J mice represent one of the most widely used mouse strains in research, these mice differ substantially from the C57BL/6N (B6/N) substrain in a number of physiological, biochemical as well as behavioral systems (11). In addition, knowledge of the phenotypic differences between the two substrains is extremely important because the B6/N substrain is increasingly used to generate null alleles for mouse genes and both substrains are widely used in genetic studies (11). Regarding the effect of HFD feeding on metabolic phenotypes in C57BL/6 mouse substrains, the B6/J substrain may have greater glucose intolerance and impaired insulin secretion compared to B6/N animals (12, 13), whereas the relative expression of NAFLD-associated phenotypes in HFD-fed B6/J and B6/N mice depends on experimental conditions such as the type of HFD used (14, 15).

Thermoneutral housing (TN; ~30°C) has previously been identified as an environmental factor promoting NAFLD progression, as demonstrated in B6/J mice fed for 24 weeks HFD with relatively low sucrose/fructose content (16). In this case, exacerbation of the disease pathogenesis was associated with an increased inflammatory response and a markedly elevated NAFLD activity score (NAS), thus capturing the characteristics of human NAFLD (16). Furthermore, the involvement of the interleukin 17 (IL-17) axis in the amplification of NAFLD caused by TN was also demonstrated (16). Interestingly, B6/N mice have been shown to secrete less IL-17 and IL-22, which originate from Th17 cells, compared to B6/J animals (17). This result, together with the aforementioned differences between B6/J and B6/N mouse substrains, prompted us to investigate how B6/N mice would respond to HFD feeding in relation to the pathogenesis of NAFLD under TN conditions.

To evaluate the effect of TN on NAFLD progression in male B6/N mice fed either a standard chow (STD) or HFD, we used comprehensive metabolipidomic profiling in addition to standard histological and molecular analyses of liver phenotypes. We demonstrated that HFD feeding greatly increases lipid deposition in the liver, but with relatively low levels of histologically proven inflammation and fibrosis, regardless of ambient temperature. In contrast, TN increased total adiposity, hepatic lipid accumulation, and NAS in STD-fed mice; however, in this case, the magnitude of the effects of TN on the hepatic phenotype was much smaller compared with the effects of HFD *per se*. The liver phenotype of mice fed STD under TN conditions was further accompanied by marked changes in the hepatic metabolome characterized by a complex decrease in various phospholipids and metabolites involved in urea cycling and oxidative stress defense. Our results may help in the selection of a suitable mouse strain for NAFLD/NASH induction experiments.

## 2 Materials and methods

### 2.1 Animals and diets

Ten-week-old male B6/N mice (Charles River Laboratories, Sulzfeld, Germany) housed one per cage were maintained at ~22°C (RT) on a 12-h light/dark cycle, with free access to water and STD (Rat/Mouse-Maintenance extrudate; ssniff Spezialdiäten GmbH, Soest, Germany; see **Supplementary Table 1** for macronutrient composition of the diets). After one week of adaptation, some of the animals were transferred to TN conditions, while the rest was kept under standard housing conditions, i.e. at RT. Half of the animals in each temperature group received HFD (product “DIO - 60 kJ% fat (Lard)”; ssniff Spezialdiäten GmbH, Soest, Germany), while the other half remained on STD.

### 2.2 Experimental setup

Mice within their temperature groups were fed STD or HFD (*n* = 8) for 24 weeks. Body weight and food consumption were recorded weekly. At week 21, tail blood was collected from mice fasted overnight to measure glycemia and plasma levels of metabolites and hormones. At week 24, mice fed *ad libitum* were killed by cervical dislocation under diethyl ether anesthesia. Liver and white adipose tissue (WAT) depots were dissected and weighed. Overall feeding efficiency was calculated by averaging the weekly feeding efficiency values obtained by dividing weight gain by energy consumed. The adiposity index was calculated as the sum of the weights of all dissected fat depots expressed as a percentage of body weight. Epididymal WAT and liver samples were fixed in formaldehyde or snap-frozen in liquid nitrogen and stored at -80°C for further analyses. Animal experiments were approved by the Institutional Animal Care and Use Committee and the Committee for Animal Protection of the Czech Academy of Sciences (Approval Number: 48/2019), in accordance with the EU Directive 2010/63/EU on the protection of animals used for scientific purposes.

### 2.3 Plasma metabolites and hormones

Plasma levels of lipid metabolites such as triglycerides (TG), non-esterified fatty acids (NEFA) and total cholesterol, and enzymes aspartate transaminase (AST) and alanine aminotransferase (ALT) were measured using the assays from Roche or Wako (for NEFA) and a Clinical Chemistry analyzer Roche/Hitachi 902 (Roche Diagnostics; Basel, Switzerland). Plasma levels of insulin and the levels of leptin and C-C motif chemokine ligand 2 (CCL2) in epididymal WAT were quantified using xMAP technology and MILLIPLEX MAP Mouse Metabolic Hormone Magnetic Bead Panel (MMHMAG-44K; Merck-Millipore). Plasma levels of total adiponectin were measured by Mouse Adiponectin ELISA kit (EZMADP-60K; Sigma-Aldrich). Fasting plasma insulin and blood glucose levels were used to quantify Homeostatic Model Assessment of Insulin Resistance (HOMA-IR), using the formula: plasma insulin (mU/L) x blood glucose (mmol/L)/22.5.

### 2.4 Immunohistochemical analysis

Liver and epididymal WAT samples were fixed in 4% formaldehyde, embedded in paraffin, and 5 µm sections were stained using hematoxylin-eosin. The NAFLD histological scoring system and NAS values (18) were used to assess the degree of NAFLD progression (18), which was evaluated by an experienced pathologist. Liver sections were also stained with picrosirius red to visualize collagen distribution, and fibrosis scores were calculated based on histological grading system (19). Macrophage accumulation in the liver was visualized using specific antibodies against F4/80 or Mac-2/galectin-3 (the latter marker was also used to detect macrophages in epididymal WAT) and the number of (hepatic) crown-like structures ((h)CLS) was counted as before (20, 21). In the region of interest on whole liver tissue slides, the present hCLS were manually counted and annotated on the digital image; they were defined as structures consisting of a hepatocyte with large lipid droplet that is ≥ 75% surrounded by F4/80 (or Mac-2/Galectin-3) positive macrophages (20). The number of hCLS was counted over a total area of 3.22 mm^2^ (equivalent to the area of six 725 × 740 μm high-power fields), with the average of six different measurements representing different areas then providing the final value for each sample. Morphometric analysis of WAT by thresholding was performed using the imaging software NIS-Elements AR3.0 (Laboratory Imaging, Prague, Czech Republic) as before (22).

### 2.5 TG content in the liver

Liver samples (~50 mg) were dissolved in 150 µL of 3M KOH (dissolved in 65% ethanol) at 70°C for 2 h. The concentration of glycerolipids was assessed as before (23).

### 2.6 Cytokine levels in WAT and liver

Frozen liver and epididymal WAT samples were homogenized in ice-cold modified RIPA buffer (50 mM Tris-HCl, pH 7.4; 150 mM NaCl; 1% NP-40) containing protease inhibitors (100 nM PMSF, 1 µg/mL leupeptin, 1 µg/mL aprotinin, and 1 µg/mL pepstatin), using an MM400 bead mill (Retsch, Haan, Germany). The homogenates were centrifuged at 13,000 x *g* for 10 min. Protein concentrations in the supernatants were determined using the BCA assay. Interleukin-1β (IL-1β), macrophage colony-stimulating factor (M-CSF), CCL2 and tumor necrosis factor (TNF)-α in the liver, and CCL2 and leptin in epididymal WAT were measured using MILLIPLEX MAP Mouse Cytokine/Chemokine Magnetic Bead Panel and MILLIPLEX MAP Mouse Adipokine Magnetic Bead Panel, respectively.

### 2.7 Gene expression analysis

Total RNA from liver (stored in RNA later; Ambion, Austin, TX, USA) and epididymal WAT was isolated using TRI Reagent (Sigma-Aldrich), and mRNA levels were measured by quantitative real-time PCR as before (23, 24). Gene names and sequences of the oligonucleotide primers are listed in **Supplementary Table 2**.

### 2.8 LC-MS analysis of liver samples

Metabolipidomic profiling of liver samples (~20 mg) was performed as before (25), using an untargeted workflow combining the lipidome, metabolome and exposome (LIMeX). Extraction was based on a biphasic solvent system of cold methanol, methyl *tert*-butyl ether, and 10% methanol. Four LC-MS platforms were used for metabolome and lipidome analysis: (i) lipidomics of complex lipids in positive ion mode, (ii) lipidomics of complex lipids in negative ion mode, (iii) metabolomics of polar metabolites in positive ion mode, and (iv) metabolomics of polar metabolites in negative ion mode. Details of sample preparation, LC-MS conditions, raw data processing and curation, and annotated metabolites can be found in Supplemental information and **Supplementary Table 3**.

### 2.9 Statistical analysis and data processing

Results are means ± SEM. To compare groups fed STD and HFD at different ambient temperatures, Two-Way ANOVA followed by Tukey *post-hoc* test was used (GraphPad Prism 9.2. software). Student’s t-test was used to analyze the effect of ambient temperature in STD-fed mice, and *p <*0.05 was considered statistically significant. Partial least-squares discriminant analysis (PLS-DA) of metabolomic data was performed using MetaboAnalyst 5.0 (26). After logarithmic transformation (base 10) and Pareto scaling, statistical models were built for metabolomic and lipidomic sets together. Exported variable importance in projection (VIP) scores were used for evaluation. Cross-validation and permutation analyses were performed on the PLS-DA models to estimate the predictive ability of the model and to minimize the risk of overfitting. Pearson’s correlation coefficient (*r*) was used to measure linear correlations between the variables.

## 3 Results

### 3.1 TN-driven changes in weight gain and adiposity in male B6/N mice are primarily observed in animals fed STD but not HFD

**Figure 1:**
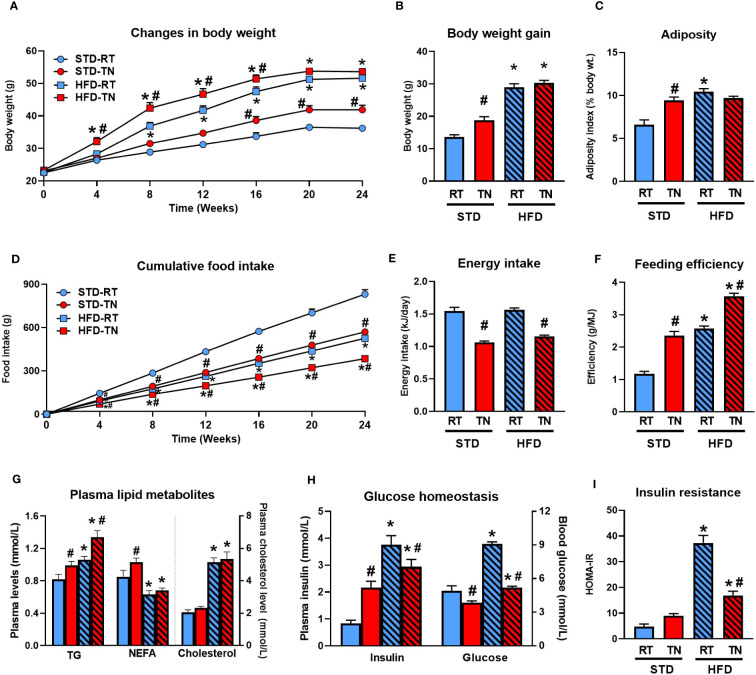
Thermoneutrality accelerates body weight gain and adiposity only in STD-fed mice. **(A)** Body weight changes during the 24-week dietary intervention. **(B)** Body weight gain. **(C)** Adiposity index (%). **(D)** Cumulative food intake. **(E)** Average daily energy intake. **(F)** Average feeding efficiency calculated over the entire duration of the experiment. **(G)** Plasma TG, NEFA and cholesterol levels in mice fasted overnight. **(H)** Plasma insulin and blood glucose levels in mice fasted overnight. **(I)** Degree of insulin resistance expressed as HOMA-IR index. Data are means ± SEM (*n* = 8). *, significant vs. respective STD; #, significant vs. the same diet at RT (Two-Way ANOVA). NEFA, non-esterified fatty acids; TG, triglycerides.

We first assessed the effect of HFD administration for 24 weeks on energy balance, adipose tissue accumulation, and plasma levels of selected metabolic markers depending on whether male B6/N mice were maintained under RT or TN conditions (see **Figure 1** and **Table 1**).

**Table 1:** Parameters of energy balance and adiposity.

	STD		HFD	
	RT	TN	RT	TN
Energy balance				
Body weight (g) – Week 0	22.5 ± 0.6	23.1 ± 0.4	22.7 ± 0.6	23.3 ± 0.7
Body weight (g) – Week 24	36.2 ± 1.0	41.9 ± 1.4^#^	51.6 ± 1.0^*^	53.6 ± 0.9^*^
Food intake (g/day)	4.97 ± 0.18	3.40 ± 0.07^#^	3.13 ± 0.06^*^	2.29 ± 0.04^*#^
WAT depots				
Epididymal WAT (g)	1.37 ± 0.16	2.27 ± 0.17^#^	2.37 ± 0.06^*^	2.11 ± 0.06
Mesenteric WAT (g)	0.59 ± 0.07	0.98 ± 0.095^#^	1.36 ± 0.05^*^	1.50 ± 0.07^*^
Subcutaneous WAT (g)	0.45 ± 0.05	0.72 ± 0.04^#^	1.67 ± 0.08^*^	1.59 ± 0.06^*^

Ten-week-old male B6/N mice were fed either STD or HFD for 24 weeks under RT or TN conditions. Data are means ± SEM (*n* = 8). ^*^Significant vs. respective STD; ^#^Significant vs. the same diet at RT; Two Way ANOVA. WAT, white adipose tissue.

Although HFD administration initially (until week 16) resulted in higher body weights in TN than RT mice (**Figure 1A**), this difference disappeared by the end of the experiment and overall weight gain was similar in both HFD-fed groups (**Table 1**). On the other hand, TN had a relatively small but significant effect on body weight gain in STD-fed mice, which became apparent from week 16 onwards (**Figures 1A, B**). Neither fat depot weights (**Table 1**) nor total adiposity (**Figure 1C**) differed between HFD-fed mice kept under RT or TN conditions, consistent with similar weight gain in these groups of animals. However, in STD-fed mice TN increased the weight of all fat depots analyzed (**Table 1**), resulting in the same overall adiposity as in both groups of HFD-fed mice (**Figure 1C**). Cumulative food intake (**Figure 1D**) as well as average daily food (**Table 1**) or energy (**Figure 1E**) intake were generally lower in mice reared under TN conditions in both dietary groups. However, the feeding efficiency was significantly higher in TN mice from both dietary groups, thus ranking the groups as follows: HFD-TN > HFD-RT = STD-TN > STD-RT (**Figure 1F**).

Compared to mice reared under RT conditions, their TN counterparts had elevated fasting plasma TG levels, regardless of diet type, whereas NEFA levels were elevated only in STD-fed mice (**Figure 1G**). As expected, plasma total cholesterol levels were markedly elevated in response to HFD feeding, with no effect of TN conditions (**Figure 1G**). In contrast, TN conditions were associated with reduced fasting glycemia, regardless of diet type (**Figure 1H**). Interestingly, plasma insulin levels in STD- and HFD-fed mice were differentially regulated by TN, with increased insulin levels observed in STD-fed mice and rather reduced levels in HFD-fed animals (**Figure 1H**). The effect of HFD feeding on the development of insulin resistance (as assessed by HOMA-IR index) was evident regardless of ambient temperature, but was much more pronounced in mice fed HFD under RT conditions (**Figure 1I**). Regardless, the above data clearly document the adaptive changes in energy homeostasis induced by TN.

### 3.2 Function of WAT is negatively affected by HFD administration regardless of ambient temperature, but signs of its dysfunction can already be observed in mice fed STD at TN

**Figure 2:**
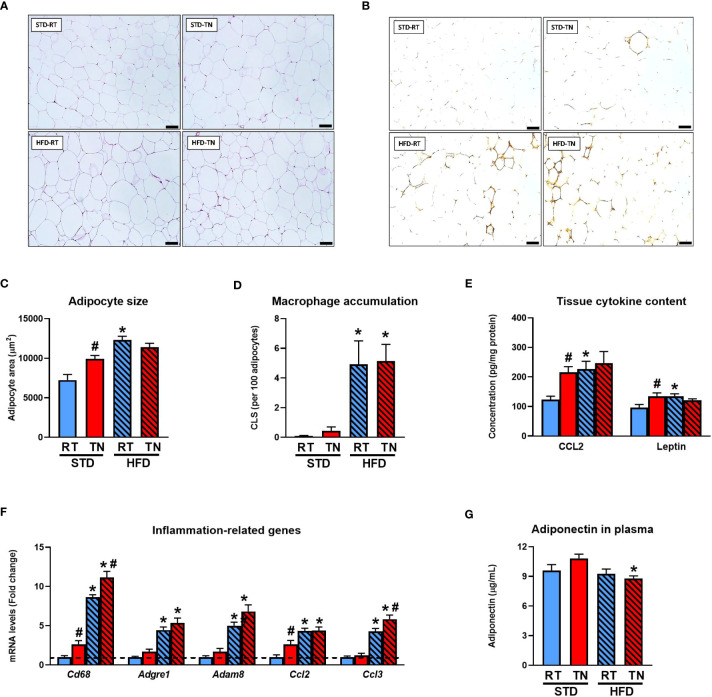
Signs of WAT dysfunction is observed in STD-fed mice kept at TN. **(A)** Sections of epididymal WAT stained with H&E. Scale bars ~100 μm. **(B)** Immunodetection of macrophage marker Mac-2/galectin-3 in epididymal WAT sections. Scale bars ~100 µm. **(C)** Adipocyte size. **(D)** Tissue macrophage content. **(E)** Inflammatory markers mRNA levels (fold change vs. STD-fed mice at RT). **(F)** Tissue content of adipokines (Note: *leptin concentrations shown in the graph are actually 100 times higher.*). **(G)** Plasma adiponectin levels. Data are means ± SEM (*n* = 6-8). *, significant vs. respective STD; #, significant vs. the same diet at RT (Two-Way ANOVA). CLS, crown-like structures; H&E, hematoxylin-eosin staining.

We analyzed the general characteristics of the epididymal WAT (**Figure 2**), as it is a major fat depot in mice and its size correlates well with the overall adiposity (**Table 1** and **Figure 1C**). Analysis of adipocyte size in hematoxylin-eosin-stained histological sections (**Figures 2A, C**) confirmed the group differences observed at the whole-tissue level. While HFD feeding, regardless of ambient temperature and consistent with adipocyte hypertrophy (**Figure 2C**), significantly increased tissue CLS numbers (**Figures 2B, D**), adipocyte hypertrophy in STD-fed TN mice was only marginally associated with increased macrophage accumulation (**Figures 2B, D**). The expression of monocyte/macrophage markers *Cd68*, *Adgre1*, *Adam8*, and chemokines *Ccl2* and *Ccl3* (see **Supplementary Table 2** for gene names) was markedly increased in HFD-fed mice, with a slight stimulatory effect of TN (**Figure 2F**). The TN environment also partially affected inflammatory gene expression in STD-fed mice, with some showing increased expression (e.g. *Cd68* and *Ccl2*) over RT-reared counterparts (**Figure 2F**). While tissue levels of CCL2 and leptin were increased by TN conditions in STD-fed animals, ambient temperature did not affect the stimulatory effect of HFD on selected cytokine levels (**Figure 2E**). Plasma levels of adiponectin, an adipokine with anti-inflammatory and insulin-sensitizing properties, showed little variation between groups, with reduced levels observed in HFD- versus STD-fed mice under TN conditions (**Figure 2G**).

### 3.3 TN conditions increased fat deposition and inflammatory profile in the livers of STD-fed mice, with less consistent effect of TN observed in HFD-fed animals

**Figure 3:**
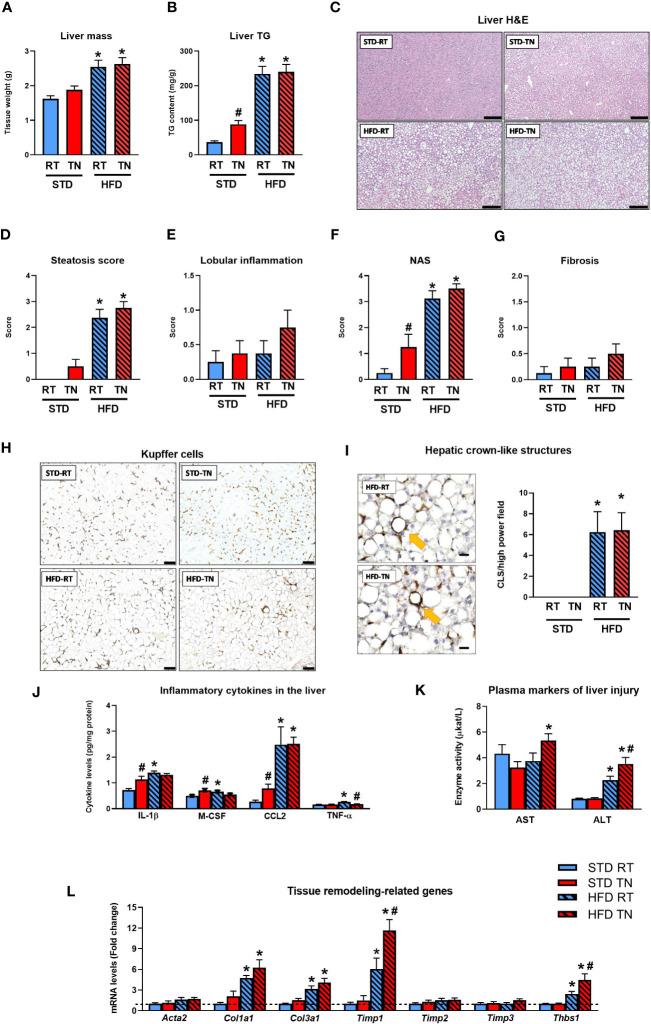
TN conditions potentiate the development of hepatic steatosis and stimulate inflammation in STD-fed mice. **(A)** Liver mass. **(B)** Liver TG content. **(C)** Hematoxylin-eosin staining of liver sections; scale bars ~200 µm. **(D)** Hepatic steatosis score. **(E)** Liver inflammation score. **(F)** NAS score. **(G)** Liver fibrosis score. **(H)** Representative liver sections showing immunodetection of the macrophage marker F4/80; scale bars ~200 µm. **(I)** Representative images of detected hCLS (shown by yellow arrows) in liver sections from mice fed HFD under RT or TN conditions (scale bars ~20 µm) and hCLS quantification in liver sections immunostained with F4/80 antibody. **(J)** Liver content of inflammatory cytokines. **(K)** Plasma AST and ALT levels. **(L)** Hepatic levels of mRNAs encoding regulators of tissue remodeling (fold change vs. STD-fed mice at RT). Data are means ± SEM (*n* = 6-8). *, significant vs. respective STD; #, significant vs. the same diet at RT (Two-Way ANOVA). H&E, hematoxylin-eosin staining; TG, triglycerides.

We further investigated the effect of ambient temperature on the development of NAFLD-related liver phenotypes in mice fed STD or HFD (**Figure 3**). While HFD administration increased liver weight regardless of ambient temperature (**Figure 3A**), biochemical analysis revealed increased liver fat accumulation not only in both temperature groups of HFD-fed animals but also in STD-fed mice in TN, albeit to a much lesser extent (**Figure 3B**). Histological analysis of hematoxylin-eosin-stained liver sections (**Figure 3C**) and assessment of the steatosis score (**Figure 3D**) were generally consistent with the results of biochemical analysis, although severe hepatic steatosis (steatosis score ~3) was observed more frequently in HFD-fed mice kept under TN (7 out of 8 animals) vs. RT (5 out of 8 animals) conditions. Besides the degree of hepatic steatosis, other components of NAS were determined, including lobular inflammation and hepatocyte ballooning. Lobular inflammation was generally low (score <1) with no significant effect of diet or temperature (**Figure 3E**). Ballooning was absent or very mild (score <0.5), with no significant differences between groups (data not shown). Consequently, NAS increased with HFD administration, regardless of ambient temperature, and to a lesser extent also in STD-fed mice with TN, which showed a significant increase in NAS compared to their counterparts housed at RT (**Figure 3F**). The degree of fibrotic changes in the liver was also relatively low in all groups, with HFD-fed mice showing only a trend toward higher fibrosis scores due to TN (**Figure 3G** and **Supplementary Figure 1**).

Immunohistochemical detection of the macrophage marker F4/80 revealed a dramatic increase in hCLS accumulation only in HFD-fed mice (**Figures 3H, I**), with no significant effect of ambient temperature. Similar results were obtained when an alternative macrophage marker, Mac-2/Galectin 3, was detected immunohistochemically in liver sections (see **Supplementary Figure 2**). Interestingly, when all F4/80-positive macrophages (i.e. mainly Kupffer cells) were quantified, including those located outside the hCLS structures, their total number did not change between groups; however, mice fed STD under RT conditions showed a trend toward decreased macrophage numbers compared with the other groups (**Supplementary Figure 3**). Regardless, hepatic levels of inflammatory cytokines such as IL-1β, M-CSF, CCL2 and TNF-α were increased in HFD- compared to STD-fed mice reared in RT (**Figure 3J**). No such effect was observed in mice with TN, mainly because most inflammatory cytokines analyzed were already elevated in STD-fed mice (**Figure 3J**). Furthermore, there was no consistent differential effect of ambient temperature on the mRNA (**Supplementary Figure 4**; see **Supplementary Table 2** for gene names) or protein (**Figure 3J**) levels of the inflammatory markers in the livers of HFD-fed mice. On the other hand, plasma markers of liver injury, particularly ALT, were elevated in HFD-fed mice, with a significant stimulatory effect of TN conditions (**Figure 3K**). Further insight was provided by gene expression analysis focusing on tissue remodeling proteins/enzymes (**Figure 3L**), whose expression was generally increased by HFD, and in some cases (e.g. *Timp1*, *Thbs1*) a significant stimulatory effect of TN (vs. RT-kept counterparts) was observed. These data in B6/N mice suggest that TN may promote fat deposition and, in part, an inflammatory response in the liver already during STD administration, whereas the deleterious effect of TN in terms of potentiating NAFLD progression in HFD-fed animals was much less evident, although HFD feeding was generally associated with more pronounced inflammatory changes in liver tissue compared with the effects of STD consumption.

### 3.4 A significant effect of ambient temperature on NAFLD-related hepatic metabolomic profiles was observed only under STD feeding conditions

**Figure 4:**
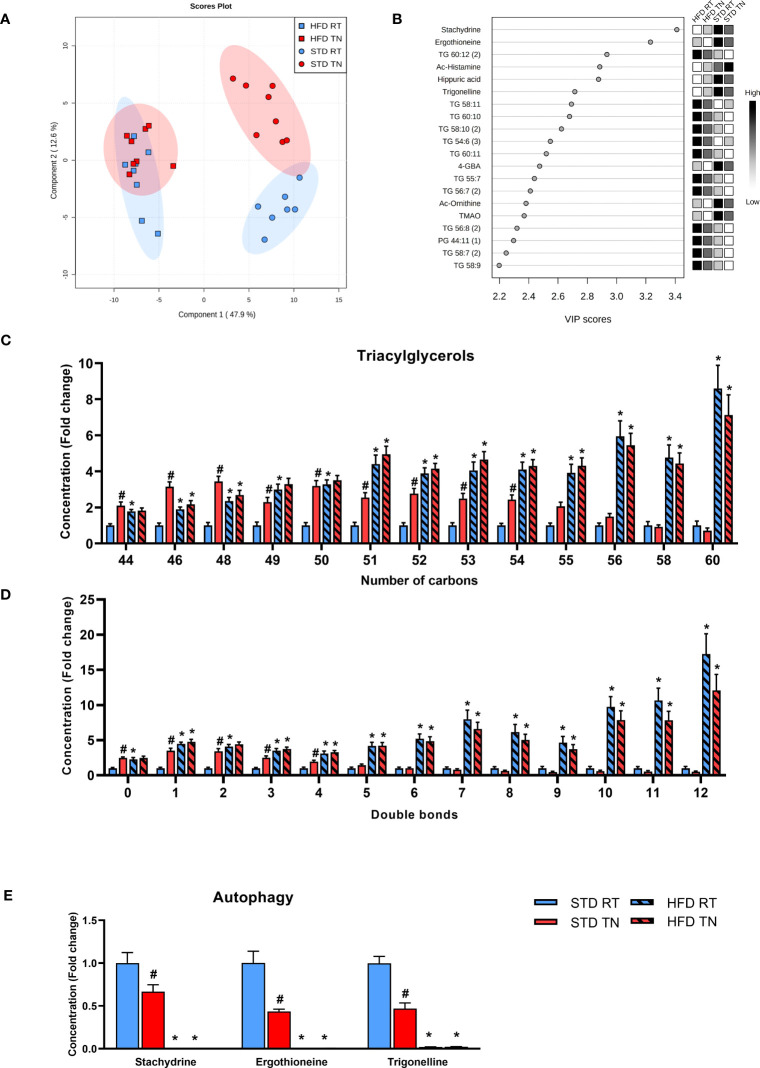
Multivariate analysis of metabolomics data from the liver reveals a distinct effect of TN only in STD-fed mice. **(A)** Four-group PLS-DA score plot based on liver metabolome. **(B)** The top 20 VIP scores identified by PLS-DA that relate to Component 1 in the PLS-DA score plot shown in panel A. **(C)** TG species grouped by number of carbon atoms per molecule. **(D)** TG species grouped by the degree of unsaturation of fatty acids in their molecule. **(E)** Autophagy-related metabolites. Data in panels C-E are shown as fold-change (vs. STD-fed mice at RT). Data are means ± SEM (*n* = 7-8). *, significant vs. respective STD; #, significant vs. the same diet at RT (Two-Way ANOVA). VIP, variable importance in projection.

To provide a global and unbiased view of diet- and temperature-induced changes in liver metabolism, we performed untargeted metabolipidomic profiling of mouse liver samples after 24 weeks of dietary intervention at different ambient temperatures. Among the normalized intensities of metabolites extracted from liver samples, a total of 549 metabolites were annotated (see **Supplementary Table 3**). Supervised multivariate analysis, i.e. PLS-DA with four-class input, exposed separation between STD-fed and HFD-fed mice regardless of ambient temperature (**Figure 4A**; see **Supplementary Figure 5** for a cross-validation of the score plots in **Figure 4A**). Interestingly, this PLS-DA further indicated temperature-driven differences in the liver metabolome of STD-fed mice (**Figure 4A**). VIP scores were calculated to define the features responsible for the measured variance in PLS-DA (**Figure 4B**). Thus, a total of 74 metabolites contributed significantly to the separation between the HFD and STD groups (i.e. within Component 1), based on a VIP score >1.5. Of the top 20 metabolites, 12 (mostly TG) were upregulated and the remaining 8 metabolites were downregulated in HFD-fed mice (**Figure 4B**). To gain insight into the diet- and temperature-dependent changes in TGs, we examined profiles of TGs sorted by either the number of carbon atoms (**Figure 4C**) or the number of double bonds (**Figure 4D**). Under RT conditions, the expected HFD-induced TG accumulation in the liver was more pronounced the longer and more unsaturated their fatty acids were. We also found a striking pattern in STD-fed mice with TN compared to all other groups, i.e. TGs with relatively low carbon number and double bond content were significantly increased. Among the polar metabolites, those associated with autophagy, such as stachydrine, ergothioneine and trigonelline, stood out with the highest VIP (**Figures 4B, E**). Not only were these autophagy-activating metabolites virtually absent in HFD-fed mice, but they were also dramatically reduced in STD-fed mice kept under TN conditions compared to their counterparts in RT (**Figure 4E**).

### 3.5 TN was associated with reduced levels of phospholipid species and metabolites involved in urea cycle and oxidative stress defense in STD-fed mice

**Figure 5:**
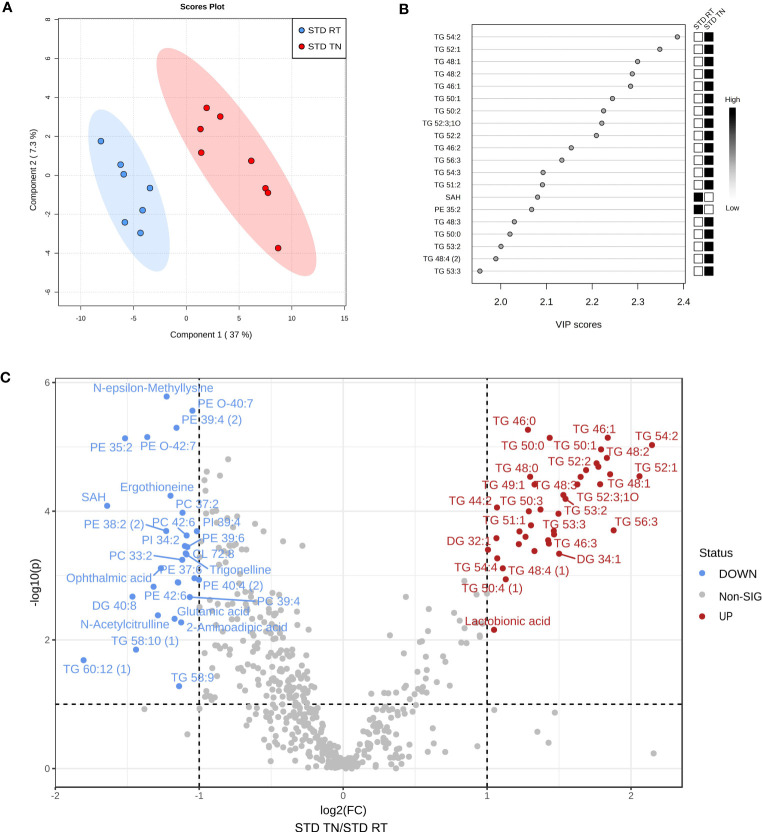
A separate analysis of liver metabolome in STD-fed mice allows identification of key factors characterizing the effect of TN. **(A)** Two-group PLS-DA score plot based on metabolomic profiles of liver. **(B)** VIP score plot for the top 20 most important metabolites identified by PLS-DA that relate to Component 1 in the PLS-DA score plot shown in panel **(A)**. **(C)** Volcano plot depicting statistical significance shown as -log10 (*p*-value) on the y-axis plotted against the magnitude of the change shown as log2 (fold-change) on the x-axis; significantly altered metabolites (TN vs. RT) are color coded.

To identify metabolic pathways separating STD-fed groups based on ambient temperature (as indicated by the four-group PLS-DA model; **Figure 4A**), we performed PLS-DA of hepatic metabolome for these two groups only (**Figure 5A**, see **Supplementary Figure 5** for a cross-validation of the score plots in **Figure 5A**). Groups of STD-fed mice kept under TN or RT conditions were primarily distinguished by alterations in TGs (**Figure 5B**). Thus, under TN conditions, animals showed significant increases in virtually all TG species measured. Metabolites of interest with high fold change and/or high statistical significance were then highlighted using a volcano plot (**Figure 5C**). Thirty-four metabolites, including several short/medium chain TGs, were increased in TN vs. RT mice, whereas 28 were decreased. Phosphatidylethanolamines (PE), phosphatidylcholines (PC), phosphatidylether-linked ethanolamines (PE-O) and several polar compounds were identified as metabolites whose levels were decreased (**Figure 5C**).

**Figure 6:**
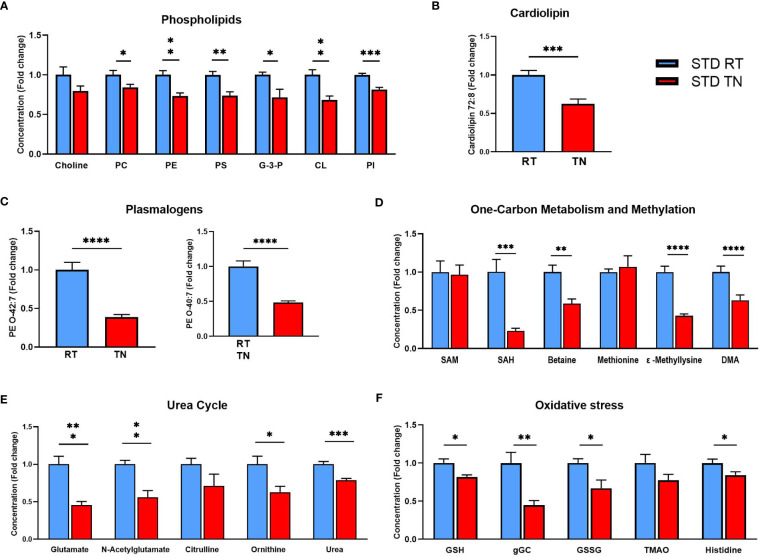
Reduced concentrations of phospholipids and metabolites related to the urea cycle and oxidative stress in the liver of STD-fed mice kept at TN. **(A)** Phospholipid levels within individual classes. **(B)** Cardiolipin CL 72:8 levels. **(C)** Plasmalogen PE O-42:7 and PE O-40:7. **(D)** Levels of metabolites related to one-carbon metabolism and methylation processes. **(E)** Levels of urea cycle-related metabolites. **(F)** Levels of oxidative stress-related metabolites. Results are shown as fold-change (vs. STD-fed mice at RT). Data are means ± SEM (*n* = 7-8). Significance: *p < 0.05; **p < 0.01; ***p < 0.001; ****p < 0.0001 (t-test). CL, cardiolipin; DMA, dimethylarginine; G-3-P, glycerol-3-phosphate; gGC, γ-glutamylcysteine; GSH, reduced glutathione; GSSG, oxidized glutathione; PC, phosphatidylcholine, PE, phosphatidylethanolamine; PI, phosphatidylinositol; PS, phosphatidylserine; SAH, S-adenosylhomocysteine; SAM, S-adenosylmethionine. TMAO, trimethylamine N-oxide.

We attempted to identify the pathways involving the most regulated metabolites to understand which biological processes are most affected by housing temperature in STD-fed animals. Besides several TG species, phospholipids were the most abundant group of regulated metabolites. Their decrease is a general consequence of TN conditions, as shown by the sums of individual phospholipid classes and their precursors (**Figure 6A**). Furthermore, concentrations of specific phospholipids known to be associated with liver health, such as cardiolipin CL 72:8 (**Figure 6B**) and plasmalogens PE O-42:7 and PE O-40:7 (**Figure 6C**), were significantly reduced in response to TN conditions. Among the polar metabolites most affected by the TN environment was S-adenosylhomocysteine (SAH; **Figure 6D**), a component of one-carbon metabolism. Levels of betaine, a methyl donor in one-carbon metabolism, were also reduced. In contrast, S-adenosylmethionine (SAM) and methionine levels were unaffected by housing temperature (**Figure 6D**). On the other hand, the methylation process, closely linked to one-carbon metabolism, was strongly reduced under TN conditions, as evidenced by reduced levels of methyllysine and dimethylarginine (DMA; **Figure 6D**). Since liver glutamate levels were reduced in mice with TN (**Figure 6D**), we examined the urea cycle and glutathione synthesis, two pathways involving glutamate. Reduced urea cycle activity due to TN was indicated by decreased levels of N-acetylglutamate, ornithine and urea (**Figure 6E**). Glutathione synthesis was similarly affected, with reduced levels of glutathione (both reduced and oxidized form) and its precursor gamma-glutamylcysteine observed in TN mice (**Figure 6F**). However, gene expression analysis involving multiple defense systems against oxidative stress (e.g. superoxide dismutase, catalase, glutathione peroxidase) did not demonstrate an effect of ambient temperature on oxidative stress in STD-fed mice (**Supplementary Figure 6**).

### 3.6 *De novo* lipogenesis may contribute to increased liver fat accumulation in STD-fed mice under TN conditions

**Figure 7:**
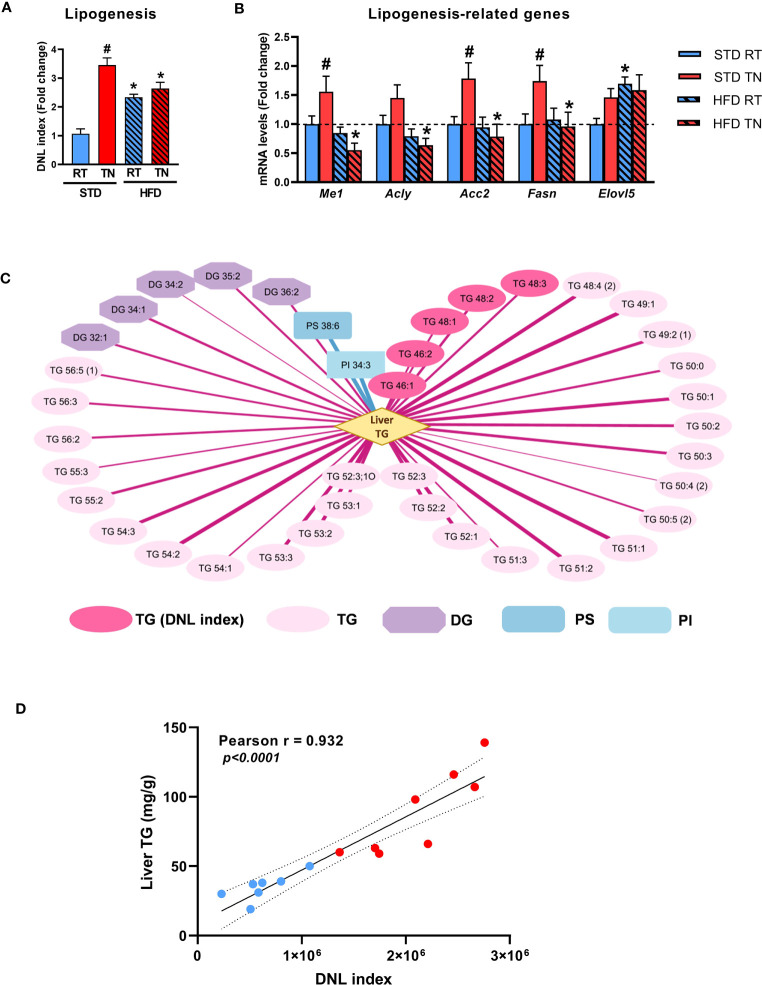
Effect of diet and ambient temperature on hepatic DNL. **(A)** DNL index. **(B)** Hepatic expression of DNL-related genes. **(C)** Strong correlations between the degree of hepatic steatosis and lipid metabolites in STD-fed mice (Pearson correlation coefficient r ≥0.85, adjusted *p* <0.05). The strength of positive (red lines) or negative (blue lines) correlation is represented by the thickness of the respective line. **(D)** Correlation between hepatic TG content and DNL index in STD-fed mice under RT (blue dots) or TN (red dots) conditions. Data are means ± SEM (*n* = 7-8). *, significant vs. respective STD; #, significant vs. the same diet at RT (Two-Way ANOVA). DG, diacylglycerol; DNL, *de novo* lipogenesis; PI, phosphatidylinositol; PS, phosphatidylserine; TG, triglyceride.

Given the marked changes in the levels of specific TG species in mouse liver (**Figure 4**), we next sought to assess the possible contribution of *de novo* lipogenesis (DNL) to hepatic steatosis. Based on the described relationship between *de novo* lipogenesis (DNL) and the amount of short-/medium-chain TGs containing up to 48 carbon atoms and 0-3 double bonds, we calculated the so-called DNL index (27). This showed the highest DNL activity in STD-fed mice under TN conditions, even compared to HFD-fed mice (**Figure 7A**). Accordingly, the mRNA levels of lipogenic genes were increased in STD-fed mice with TN (**Figure 7B**). Correlation analysis then revealed a strong positive correlation (r ≥0.85, *p <*0.05) between fat accumulation in the liver of STD-fed mice and various TG species, including those constituting the DNL index, as well as some diacylglycerols (DG). Only a few metabolites, such as the phospholipids PS 38:6 and PI 34:3, were negatively correlated (**Figure 7C**). Moreover, when only TG species representing the DNL index were considered, the value of their positive association with liver TG levels was r = 0.932 (**Figure 7D**). No such relationship was observed in HFD-fed mice (**Supplementary Figure 7**). Our results in B6/N mice show that TN conditions accelerate increased fat accumulation in the liver of STD-fed mice, and one of the mechanisms involved in this process is likely to be activated DNL.

## 4 Discussion

The main objective of this study was to analyze the TN-driven progression of NAFLD in B6/N mice, one of the widely used substrains of C57BL/6 mice. In male mice fed a lard-based HFD for 24 weeks, we show that although HFD feeding significantly promoted fat deposition and generally increased the expression of inflammatory and tissue remodeling markers in the liver, TN conditions did not have consistent stimulatory effects on these phenotypes and did not promote the development of histologically proven NASH. In contrast, in STD-fed mice TN promoted weight gain and negatively affected both WAT and liver properties, as manifested by increased adiposity, plasma lipid levels, adipocyte hypertrophy and WAT inflammation, as well as hepatic steatosis. Although metabolomic profiling in the liver confirmed the dominant role of autophagy-related metabolites in the negative effects of HFD, the effect of TN was mainly demonstrated in STD-fed mice, where a specific pattern of metabolite changes characterized by a complex decrease of various phospholipid species and a reduction of metabolites involved in urea cycle and oxidative stress response was observed.

We attempted to approximate as closely as possible the experimental design of the previous study investigating the effect of TN at 30°C on NAFLD progression in B6/J mice (16). In addition to the same study duration of 24 weeks, we used a commercial HFD of a similar composition in terms of fat content (~35% by weight; mostly lard), combined with a relatively low cholesterol (~0.02-0.03%) and sucrose (~9%) contents. Despite these similarities, our study did not reveal a major amplifying effect of TN on NAFLD progression in HFD-fed B6/N mice, particularly for the development of NASH. The increase in NAS generally observed in HFD-fed animals was not affected by housing temperature. Although there was a nonsignificant trend toward an increase in the lobular inflammation scores in HFD-fed mice under TN conditions (vs. RT-kept counterparts), the overall inflammation score was low and did not exceed the average value of ~0.75. In addition to using a standard histological scoring system to assess the degree of inflammation and/or fibrosis (18, 19), we also performed immunodetection of two different macrophage markers (Mac-2/Galectin-3 and F4/80) and quantified the number of hCLS in liver sections (20, 21). The number of hCLS detected was very similar regardless of which macrophage marker was used, and their number was significantly increased only in HFD-fed mice, with no effect of ambient temperature. On the other hand, liver sections stained with F4/80 antibody have a different appearance than sections stained with Mac-2/Galectin-3 antibody because Mac-2/Galectin-3 is primarily associated with activated macrophages that enter the liver from the peripheral circulation (28), whereas F4/80 is highly expressed on resident macrophages (i.e. Kupffer cells (29)). Nonetheless, the massive accumulation of hepatic lipids and steatosis scores of ~3 were the main contributors to the elevated NAS values in HFD-fed mice in our study. In contrast, in the previous study (16) NAS values for B6/J males fed HFD under TN conditions were ~7, while liver TG content reached a maximum of only ~120 mg/g tissue, i.e. it was almost twice lower than in our study. Different degrees of NAFLD progression observed in HFD-fed mice in these studies could be related to the genetic background of the mice used. Indeed, Kawashita et al. (15) recently observed a dramatic increase in liver inflammation scores and increased expression of inflammatory markers CCL2 and TNF-α in B6/J but not B6/N mice fed HFD for 30 weeks. Likewise, liver regeneration and a rapid inflammatory response was observed in B6/N mice compared to B6/J mice during acute exposure to carbon tetrachloride (30). In addition, a mutation in the gene for *nicotinamide nucleotide transhydrogenase*, which has been found exclusively in B6/J mice (31), may be involved in their greater inflammatory response compared to B6/N animals, as impaired conversion of NADP^+^ to NADPH may negatively affect the detoxification of reactive oxygen species. Thus, our results support the notion that genetic makeup combined with the type, absolute amount and relative proportion of key dietary components may influence NASH development (5, 6), as well as the severity of the metabolic syndrome (32).

Interestingly, the weight gain and adiposity induced by TN in STD-fed mice can be attributable to a ~2-fold increase in feeding efficiency in these animals (vs. RT-kept counterparts). In contrast, the lack of effect of TN on body weight gain in HFD-fed mice is consistent with a previous study by Giles et al. (16) in which the stimulatory effect of TN on body weight gain in HFD-fed mice disappeared towards the end of the study. Although another study found slightly increased weight gain in HFD-fed mice under TN conditions (33), that study was conducted in A/J mice, which are known to be relatively resistant to diet-induced obesity (34). Regardless, alterations in gut microbiota and intestinal barrier function may also be involved in promoting inflammation and progression of obesity-related NAFLD (35), and TN has been shown to cause dysbiosis (i.e. expansion of the phylum *Bacteroidetes*) and augment intestinal permeability in HFD-fed B6/J mice (16, 36). Microbiome analysis revealed some differences between B6/J and B6/N mouse substrains in cecal and fecal microbial communities (37, 38), and there is considerable evidence that the phenotypes of mouse models can vary quite a bit between animal facilities and that these differences may be related to differences in the microbiota (39). Therefore, an attenuated response to HFD administration and/or TN conditions in terms of negative modulation of gut function could explain the lack of significant liver inflammation observed in HFD-fed B6/N mice in our study. On the other hand, impaired WAT function may have negatively affected liver function and contributed to NAFLD development in STD-fed mice under TN conditions, as healthy adipose tissue is essential for the function of extra-adipose organs, including the liver (40, 41).

We used metabolomic analysis to further elucidate mechanisms responsible for observed differences in liver steatosis. Our primary focus was on analyzing the metabolic changes associated with i) the overall effect of HFD versus STD feeding at a given ambient temperature, and ii) increased liver steatosis in STD-fed mice under TN conditions. Among the key metabolites distinguishing STD-fed mice from HFD-fed mice was a group of polar metabolites such as stachydrine, ergothioneine, and trigonelline, microbiota products with documented beneficial effects, including autophagy induction and antioxidant activity (42–44). This substantial reduction in autophagy-regulating metabolites may contribute to the impairment of the autophagosomal-lysosomal system, as long-term HFD administration has been shown to negatively affect autophagosome assembly, autophagosome-lysosome fusion, and lysosome function (45). Although their decline may indicate changes in the gut microbiota composition associated with HFD feeding, it reflects rather the absence of these substances in the HFD (42–44). Similarly, the reduced levels of these bioactive substances in the livers of STD-fed mice under TN conditions may reflect their lower dietary intake. However, it is not entirely clear how these alkaloids contribute to the development of hepatic steatosis and/or NAFLD progression.

TGs are another group of metabolites that contribute to the separation between STD-fed and HFD-fed mice, as well as to the distinction between the two STD-fed groups. Whereas HFD-fed mice accumulated mainly long-chain TGs with a high number of double bonds, suggesting increased dietary fatty acid intake, STD-fed mice with TN showed a profile with a predominance of short-chain TGs and a low number of double bonds, indicative of active DNL (27). In fact, increased DNL activity, which we also confirmed at the level of lipogenic gene expression, could represent an important factor contributing to the development of TN-induced hepatic steatosis in STD-fed mice. The mechanism of DNL induction in these mice may involve activation of the transcription factor sterol regulatory element-binding protein 1c, a master regulator of the fatty acid biosynthetic pathway (46), probably due to elevated plasma insulin levels in TN conditions. Increased insulin levels in STD-fed and TN-reared mice have been observed previously (47) and may indicate increased metabolic demands on the pancreas for insulin production and secretion under TN conditions, perhaps as an adaptive response to impaired insulin sensitivity. However, other mechanisms that contribute to fat accumulation in the liver of STD-fed mice cannot be ruled out. For example, the elevated circulating levels of NEFA observed in these animals may also be involved in the development of hepatic steatosis, similar to that seen in NAFLD patients (48).

It is known that the development of NAFLD and its further progression are also associated with changes in one-carbon metabolism (49). Hyperhomocysteinemia, one of the key markers of impaired one-carbon metabolism (50), is inherent in many models of NAFLD, e.g. in mice consuming a diet deficient or rich in methionine (51). Surprisingly, SAH was reduced in the liver of our mice in response to both HFD and TN, but their effects were not additive. Simultaneously, the amount of betaine, a one-carbon methyl donor known to be hepatoprotective (52), was also reduced under HFD or TN conditions. Despite the reduction in betaine, essential for remethylating homocysteine to methionine (53), levels of SAM and its precursor methionine remained unchanged in the study groups. Although the levels of these key metabolites of one-carbon metabolism seem to be preserved, we observed changes in several downstream processes such as protein methylation and urea cycle. These metabolic processes were equally reduced under HFD or TN conditions. Reduced urea cycle activity in patients with NAFLD leads to elevated ammonia levels, which are further increased in patients with NASH (54) and can lead to tissue scarring, increasing the risk of disease progression (55). Oxidative stress, on the other hand, could be one of the main pathophysiological consequences of the abnormal functioning of one-carbon metabolism in STD-fed mice in TN, as evidenced by reduced levels of metabolites of the glutathione system. Alternatively, reduced glutathione levels may rather indicate reduced availability of precursors such as glutamate, cysteine and glycine (56). Glutathione levels were unaltered in HFD-fed mice (**Supplementary Figure 6**), suggesting that the transsulfuration pathway is preserved in these animals. Thus, in our study, the absence of further impairment of urea cycle and one-carbon metabolism, together with preserved oxidative stress defenses, may explain the lack of acceleration of NAFLD progression in HFD-fed mice under TN conditions.

Considering the limitations of our study, we did not investigate the effect of TN on the development of NAFLD/NASH in female mice. Thus, we cannot generalize our results with respect to potential sex differences, which is important for the design and planning of future studies conducted in C57BL/6N mice. In addition, the type of dietary stimulus used to induce NAFLD/NASH in our study was identical to that used in the original study by Giles et al. (16), i.e. lard was the main source of lipids and the sucrose (fructose) content was relatively low. High-fructose diets are known to promote NASH (5, 6), even in mice of the C57BL/6N background (57). Therefore, we do not know whether TN conditions would promote NAFLD progression if a different type of HFD with a higher sucrose content or HFD combined with fructose/glucose in the drinking water were used.

In conclusion, TN combined with long-term administration of a lard-based low-sucrose HFD has a relatively mild stimulatory effect on NAFLD progression in male B6/N mice. Although HFD feeding promoted the development of an inflammatory phenotype in the liver compared with STD-fed mice, the effect of ambient temperature on the expression of inflammatory markers was inconsistent and did not manifest in a substantial degree of histologically proven NASH. In contrast, the development of hepatic steatosis and apparent disruption of the hepatic metabolome are characteristic of B6/N mice fed STD under TN conditions, which may potentially serve as a model for investigating early pathophysiological changes associated with the development and progression of NAFLD. There is probably no ideal mouse model of diet-induced progressive NAFLD that faithfully mimics the disease in obese humans. While the original findings that TN potentiates NAFLD progression were made in B6/J mice, our somewhat dissimilar findings in B6/N mice fed the same HFD may have implications for the design of future studies targeting NAFLD/NASH development *via* modulation of ambient temperature.

## Data availability statement

The raw data that support the conclusions of this article will be made available by the corresponding author upon reasonable request without undue reservation.

## Ethics statement

The animal study was reviewed and approved by Institutional Animal Care and Use Committee and the Committee for Animal Protection of the Czech Academy of Sciences (Approval Number: 48/2019), in accordance with the EU Directive 2010/63/EU on the protection of animals used for scientific purposes.

## Author contributions

MR conceived and designed research; OH, GS, VK, KB, MM, TC, II, PJ, KL, and MR performed experiments; OH, GS, VK, KB, MM, TC, II, PJ, KL, and MR analyzed data; OH, GS, KB, TC, and MR interpreted results of experiments; OH, GS, and MR prepared figures; OH and MR drafted the manuscript; OH, GS, VK, KB, TC, PJ, KL, JK, and MR edited and revised the manuscript. All authors read and approved the final paper.
