# Supplementary material for: Thermoneutral housing promotes hepatic steatosis in standard diet-fed C57BL/6N mice, with a less pronounced effect on NAFLD progression upon high-fat feeding
Source: Front Endocrinol (Lausanne). 2023 Jul 12;14:1205703. doi: 10.3389/fendo.2023.1205703 (PMC10369058; doi:10.3389/fendo.2023.1205703)
Supplement: Supplementary file 1 [file DataSheet_1.docx]

Supplementary Material

Thermoneutral housing potentiates NAFLD progression in C57BL/6N mice fed standard but not high-fat diet

Olga Horakova, Gabriella Sistilli, Veronika Kalendova, Kristina Bardova, Marko Mitrovic, Tomas Cajka, Ilaria Irodenko, Petra Janovska, Karoline Lackner, Jan Kopecky and Martin Rossmeisl*

*** Correspondence:** Martin Rossmeisl: [martin.rossmeisl@fgu.cas.cz](mailto:martin.rossmeisl@fgu.cas.cz)

Table of contents
1. Supplementary methods................................................................................................2
2. Supplementary figures..................................................................................................5
3. Supplementary tables....................................................................................................14

# Supplementary methods

*Sample extraction for metabolomics and lipidomics analyses of the liver.* Liver samples (20 mg) were homogenized with 275 µL methanol containing internal standards (PE 17:0/17:0, PG 17:0/17:0, LPC 17:1, Sphingosine d17:1, Cer d18:1/17:0, SM d18:1/17:0, PC 15:0/18:1-d7, cholesterol-d7, TG 17:0/17:1/17:0-*d*_5_, DG 12:0/12:0/0:0, DG 18:1/2:0/0:0, LPE 17:1, oleic acid-*d*_9_, PI 15:0/18:1-*d*_7_, MAG 17:0/0:0/0:0, PS 17:0/17:0, HexCer d18:1/17:0, DG 18:1/0:0/18:1-*d*_5_, TG 20:0/20:1/20:0-*d*_5_, LPG 17:1, LPS 17:1, cardiolipin 16:0/16:0/16:0/16:0) and 275 µL 10% methanol containing internal standards (caffeine-*d*_9_, acetylcholine-*d*_4_, creatinine-*d*_3_, choline-*d*_9_, TMAO-*d*_9_, *N*-methylnicotinamide-*d*_4_, betaine-*d*_9_, butyrobetaine-*d*_9_, creatine-*d*_3_, cotinine-*d*_3_, glucose-*d*_7_, succinic acid-*d*_4_, metformin-*d*_6_) for 1.5 min using a grinder (MM400, Retsch, Germany). Then, 1 mL of MTBE with internal standard (CE 22:1) was added, the tubes were shaken for 1 min and centrifuged at 16,000 rpm for 5 min.

For lipidomic profiling, 100 µL of the upper organic phase was collected, evaporated, and resuspended using 500 µL methanol with internal standard (CUDA), shaken for 30 s, centrifuged at 16,000 rpm for 5 min, and used for LC-MS analysis. An aliquot of 70 µL of the bottom aqueous phase was collected, evaporated, resuspended in 70 µL of an acetonitrile/water (4:1, *v*/*v*) mixture with internal standards (CUDA and Val-Tyr-Val), shaken for 30 s, centrifuged at 16,000 rpm for 5 min and analyzed using HILIC metabolomics platform. Another 70 µL of the bottom aqueous phase was mixed with 210 µL of an isopropanol/acetonitrile (1:1, *v*/*v*) mixture, shaken for 30 s, centrifuged at 16,000 rpm for 5 min, and the supernatant was evaporated, resuspended in 5% methanol/0.2% formic acid with internal standards (CUDA and Val-Tyr-Val), shaken for 30 s, centrifuged at 16,000 rpm for 5 min and analyzed using HSS T3 metabolomics platform.

*LC-MS-based lipidomics.* The LC-MS systems consisted of a Vanquish UHPLC System (Thermo Fisher Scientific, Bremen, Germany) coupled to a Q Exactive Plus mass spectrometer (Thermo Fisher Scientific, Bremen, Germany). Lipids were separated on an Acquity UPLC BEH C18 column (50 × 2.1 mm i.d.; 1.7 μm particle size) coupled to an Acquity UPLC BEH C18 VanGuard pre-column (5 × 2.1 mm i.d.; 1.7 μm particle size) (Waters, Milford, MA, USA). The column was maintained at 65 °C at a flow-rate of 0.6 mL/min. For LC-ESI(+)-MS analysis, the mobile phase consisted of (A) 60:40 (*v*/*v*) acetonitrile:water with ammonium formate (10 mM) and formic acid (0.1%) and (B) 90:10:0.1 (*v*/*v*/*v*) isopropanol:acetonitrile:water with ammonium formate (10 mM) and formic acid (0.1%). For LC-ESI(−)-MS analysis, the composition of the solvent mixtures was the same except for the addition of ammonium acetate (10 mM) and acetic acid (0.1%) as mobile-phase modifiers. Separation was conducted under the following gradient for LC-ESI(+)-MS: 0 min 15% (B); 0–1 min 30% (B); 1–1.3 min from 30% to 48% (B); 1.3–5.5 min from 48% to 82% (B); 5.5–5.8 min from 82% to 99% (B); 5.8–6 min 99% (B); 6–6.1 min from 99% to 15% (B); 6.1–7 min 15% (B) + 1 min preinjection steps. For LC-ESI(−)-MS, the following gradient was used: 0 min 15% (B); 0–1 min 30% (B); 1–1.3 min from 30% to 48% (B); 1.3–4.8 min from 48% to 76% (B); 4.8–4.9 min from 76% to 99% (B); 4.9–5.3 min 99% (B); 5.3–5.4 min from 99% to 15% (B); 5.4–6.3 min 15% (B) + 1 min preinjection steps. A sample volume of 0.5 and 3 μL was used for the injection in ESI(+) and ESI(−), respectively. The sample temperature was maintained at 4 °C.

The ESI source and MS parameters were: sheath gas pressure, 60 arbitrary units; aux gas flow, 25 arbitrary units; sweep gas flow, 2 arbitrary units; capillary temperature, 300 °C; aux gas heater temperature, 370 °C; MS1 mass range, *m*/*z* 200–1700; MS1 resolving power, 35,000 FWHM (*m/z* 200); number of data-dependent scans per cycle, 3; MS/MS resolving power, 17,500 FWHM (*m/z* 200). For ESI(+), a spray voltage of 3.6 kV and normalized collision energy of 20% was used; for ESI(−), a spray voltage of −3.0 kV and normalized collision energy of 10, 20 and 30% were set up.

*LC-MS-based metabolomics.* Polar metabolites were separated on an Acquity UPLC BEH Amide column (50 × 2.1 mm i.d.; 1.7 μm particle size) coupled to an Acquity UPLC BEH Amide VanGuard pre-column (5 × 2.1 mm i.d.; 1.7 μm particle size) (Waters, Milford, MA, USA). The column was maintained at 45 °C at a flow-rate of 0.4 mL/min. The mobile phase consisted of (A) water with ammonium formate (10 mM) and formic acid (0.125%) and (B) acetonitrile:water (95/5) with ammonium formate (10 mM) and formic acid (0.125%). Separation was conducted under the following gradient: 0 min 100% (B); 0–1 min 100% (B); 1–3.9 min from 100% to 70% (B); 3.9–5.1 min from 70% to 30% (B); 5.1–6.4 min from 30% to 100% (B); 6.4–7.5 min 100% (B) + 1 min preinjection steps. A sample volume of 0.5 μL was used for the injection in ESI(+). The sample temperature was maintained at 4 °C.

Polar metabolites were also separated on an Acquity UPLC HSS T3 column (50 × 2.1 mm i.d.; 1.8 μm particle size) coupled to an Acquity UPLC HSS T3 VanGuard pre-column (5 × 2.1 mm i.d.; 1.8 μm particle size) (Waters, Milford, MA, USA). The column was maintained at 45 °C using a ramped flow-rate. The mobile phase consisted of (A) water with formic acid (0.2%) and (B) methanol with formic acid (0.1%). Separation was conducted under the following gradient: 0 min 1% (B) 0.3 mL/min; 0–0.5 min 1% (B) 0.3 mL/min; 0.5–2 min from 1% to 60% (B) 0.3 mL/min; 2–2.3 min from 60% to 95% (B) from 0.3 mL/min to 0.5 mL/min; 2.3–3.0 min 95% (B) 0.5 mL/min; 3.0–3.1 min from 95% to 1% (B) 0.5 mL/min; 3.1–4  min 1% (B) 0.5 mL/min; 4–4.1 min 1% (B) from 0.5 mL/min to 0.3 mL/min; 4.1–4.5 min 1% (B) 0.3 mL/min + 1 min preinjection steps. A sample volume of 5 μL was used for the injection in ESI(−). The sample temperature was maintained at 4 °C.

The ESI source and MS parameters were: sheath gas pressure, 50 arbitrary units; aux gas flow, 13 arbitrary units; sweep gas flow, 3 arbitrary units; capillary temperature, 260 °C; aux gas heater temperature, 425 °C; MS1 mass range, *m/z* 60–900; MS1 resolving power, 35,000 FWHM (*m/z* 200); number of data-dependent scans per cycle, 3; MS/MS resolving power, 17,500 FWHM (*m/z* 200). A spray voltage of 3.6 kV and −2.5 kV for ESI(+) and ESI(−) were used. A normalized collision energy of 20, 30 and 40% for all metabolomics platforms was used.

*Quality control.* Quality control was assured by (i) randomization of the actual samples within the sequence, (ii) injection of quality control (QC) pool samples at the beginning and the end of the sequence and between each 10 actual samples, (iii) analysis of procedure blanks, (iv) serial dilution of QC sample (0, 1/16, 1/8, 1/4, 1/2, 1), (v) checking the peak shape and the intensity of spiked internal standards and the internal standard added prior to injection.

*Data processing.* LC-MS data from metabolomic and lipidomic profiling were processed through MS-DIAL v. 3.90 software. Metabolites were annotated using in-house retention time–*m*/*z* library and MS/MS libraries available from commercial and open sources (NIST17, MassBank, MoNA). Lipids were annotated using LipidBlast in-built in MS-DIAL [1]. Raw data were filtered using blank samples, serial dilution samples, and QC pool samples with relative standard deviation (RSD) <30%, and then normalized using locally estimated scatterplot smoothing (LOESS) approach by means of QC pool samples injected regularly between 10 actual samples followed by sample-weight normalization. Data were exported as the detector signal intensity in arbitrary units (A.U.).

**References**

[1] Tsugawa, H.; Cajka, T.; Kind, T.; Ma, Y.; Higgins, B.; Ikeda, K.; Kanazawa, M.; VanderGheynst, J.; Fiehn, O.; Arita, M. MS-DIAL: data-independent MS/MS deconvolution for comprehensive metabolome analysis. *Nature Methods* **2015**, 12, 523-526, doi: 10.1038/nmeth.3393.

#
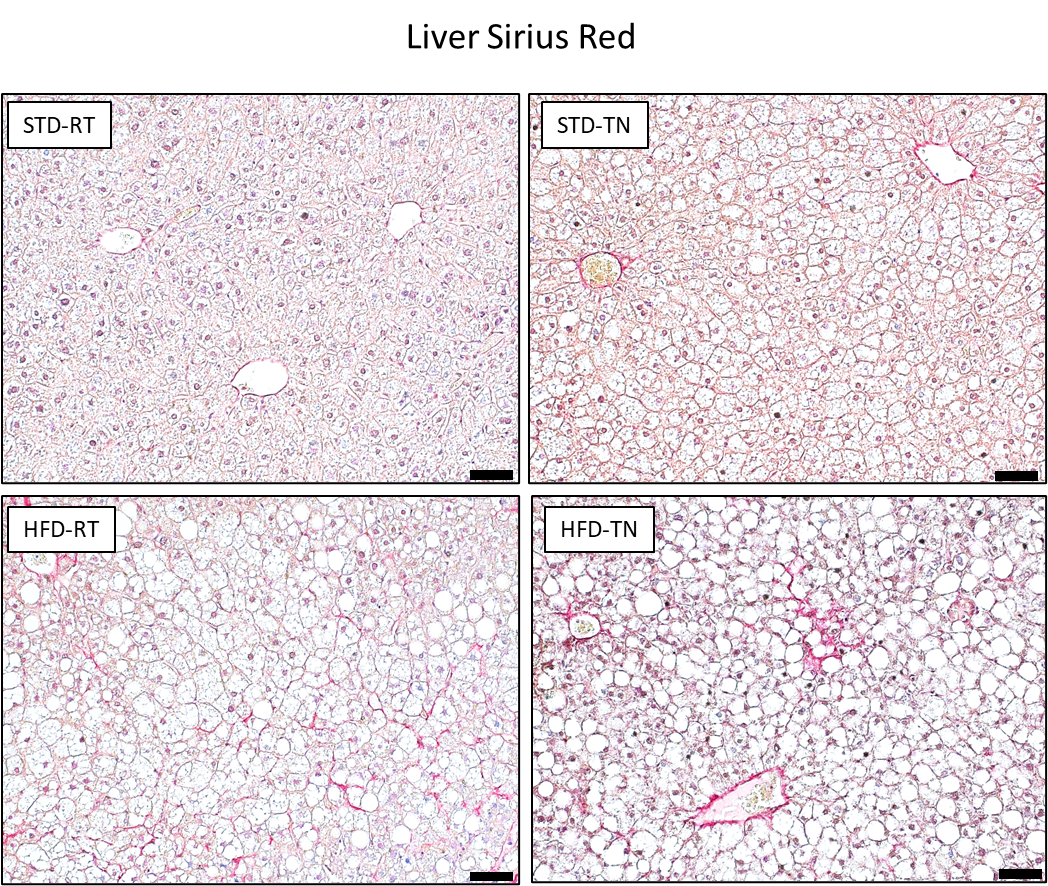
Supplementary Figures

**Supplementary Figure 1.** Thermoneutral housing (TN) conditions do not significantly potentiate the development of fibrosis in the livers of male C57BL/6N mice fed standard diet (STD) or high-fat diet (HFD), compared to mice kept under standard room temperature (RT). Representative sections of liver stained with Sirius Red dye; scale bars ~200 µm.


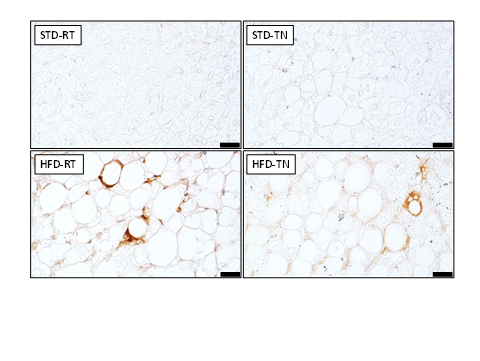


Liver Mac-2/Galectin-3

A

B

**
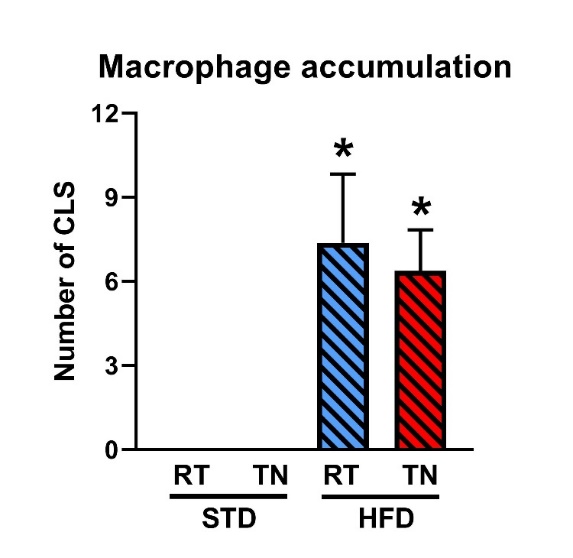
**

**Supplementary Figure 2.** Macrophage content in the liver was markedly increased in mice fed a high-fat diet (HFD) compared to standard diet (STD)-fed mice, without any differential effect of thermoneutral housing (TN) vs. standard room temperature (RT). **(A)** Accumulation of activated macrophages in the liver visualized by immunohistochemical detection of the macrophage marker Mac-2/galectin-3; scale bars ~20 µm. **(B)** Quantification of hepatic crown-like structures (CLS) calculated over a total area of 3.22 mm^2^ (equivalent to the area of six 725 x 740 µm high-power fields), with the average of six different measurements representing different areas then providing the final value for each sample. Data are expressed as means ± SEM (*n* = 7-8). *, significant effect of HFD (vs. respective STD; Two-Way ANOVA).


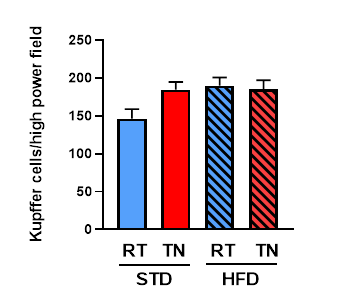


**Supplementary Figure 3**. Tissue content of F4/80 positive macrophages (i.e. Kupffer cells) in the liver. Their content was similar in mice fed a high-fat diet (HFD) compared to those fed a standard diet (STD), with no difference in the effect of thermoneutral housing (TN) compared to standard room temperature (RT) conditions. Data are expressed as means ± SEM (*n* = 7-8). (Two-Way ANOVA).


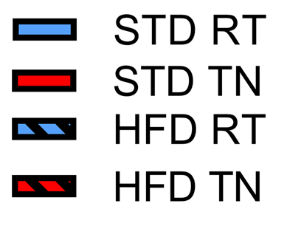

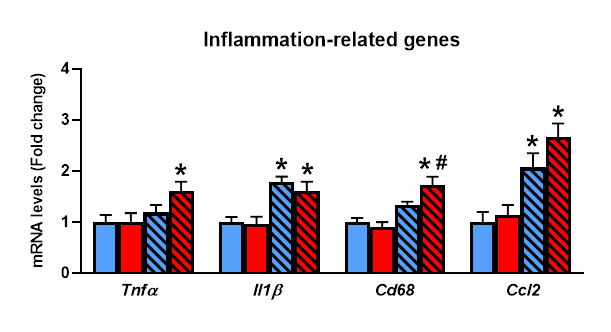


**Supplementary Figure 4.** Expression of inflammatory genes in the liver was generally increased in response to high-fat diet (HFD) feeding compared to standard diet (STD)-fed mice, with a modest stimulatory effect of thermoneutral housing (TN) compared to standard room temperature (RT). The levels of mRNAs encoding inflammatory markers are shown as fold change (vs. STD-fed mice at RT; set to 1). Data are expressed as means ± SEM (*n* = 7-8). *, significant effect of HFD (vs. respective STD); #, significant effect of TN (vs. the same diet at RT; Two-Way ANOVA).


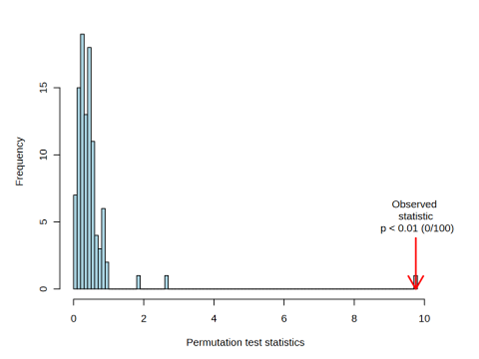

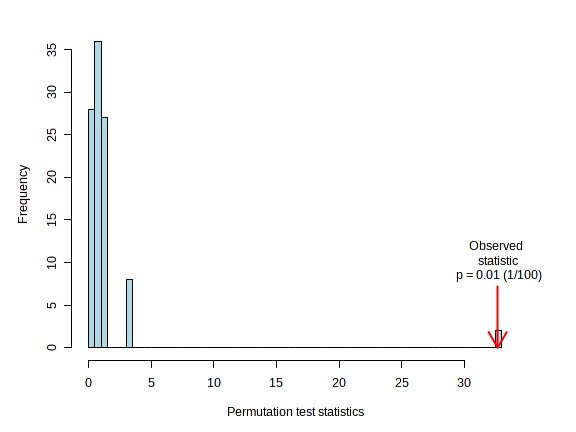

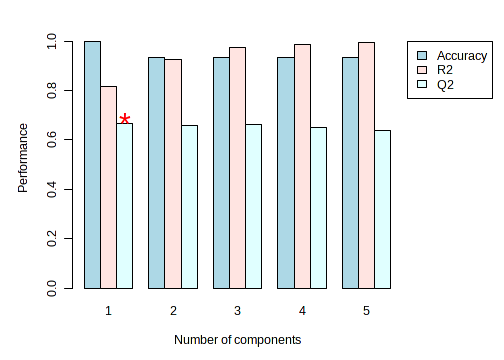

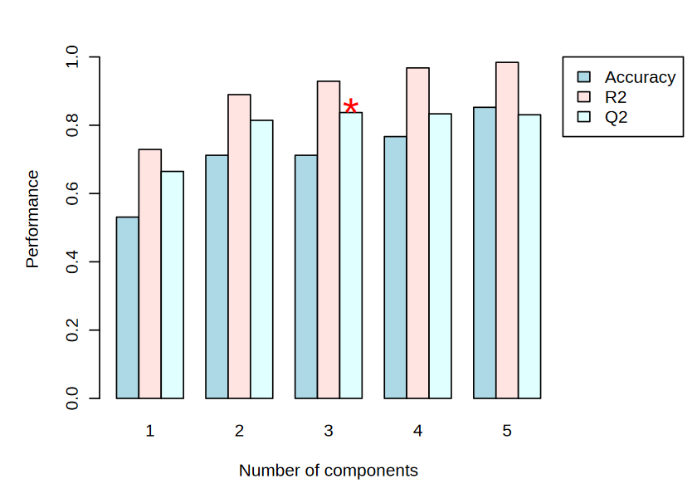


B

D

C

A

**Supplementary Figure 5.** Validation of Partial least squares Discriminant Analysis (PLS-DA) models. Plots obtained by: **A** cross-validation method, and **B** the permutation test (100 times), applied on data from PLS-DA model of 4 groups (corresponding to PLS-DA in Figure 4). Plots obtained by: **C** cross-validation method, and **D** the permutation test (100 times), applied on data from PLS-DA model of 2 groups of STD-fed mice (corresponding to PLS-DA in Figure 5). PLS-DA cross-validation details: 5-fold CV. Permutation test details: Separation distance (B/W).

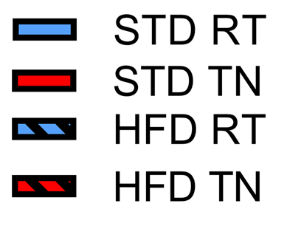


**Supplementary Figure 6.** The expression of genes related to oxidative stress in the liver was generally unchanged in response to feeding a high-fat diet (HFD) compared to mice fed a standard diet (STD), with the exception of *Gpx1* and *Nrf2*, whose expression was stimulated to some extent by feeding an HFD compared to mice fed an STD. The effect of thermoneutral housing (TN) compared to standard room temperature (RT) was not observed. The levels of mRNAs are shown as fold change (vs. STD-fed mice at RT; set to 1). Data are expressed as means ± SEM (n = 7-8). *, significant effect of HFD (vs. respective STD; Two-Way ANOVA).


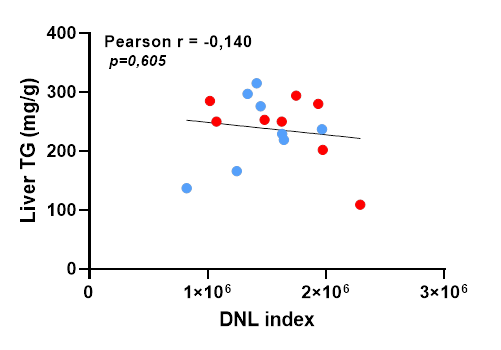


**Supplementary Figure 7.** In mice fed a high-fat diet (HFD) at standard room temperature (RT; blue dots) or at thermoneutrality (TN; red dots), no relationship was found between TG content in the liver and the DNL index value (i.e., the sum of all TG species relevant for the calculation of this index; see main article). DNL, *de novo* lipogenesis.

**Supplementary Figure 8.** Concentrations of metabolites related to phospholipid metabolism, oxidative stress, one-carbon metabolism and urea cycle in the liver of experimental animals. **A** Phospholipid levels within individual classes. **B** Cardiolipin 72:8 levels. **C** Plasmalogen PE O-42:7 and PE O-40:7 levels. **D** Levels of metabolites related to one-carbon metabolism and methylation processes. **E** Levels of urea cycle-related metabolites. **F** Levels of oxidative stress-related metabolites. Results are shown as fold-change (vs. STD-fed mice at RT). Data are means ± SEM (*n* = 7-8). *, significant effect of HFD (vs. respective STD); #, significant effect of TN (vs. the same diet at RT; Two-Way ANOVA).

# Supplementary Tables

**Supplementary Table 1** Diet composition

|  | **STD** | **HFD** |
| --- | --- | --- |
| **Company** | ssniff Spezialdiäten GmbH | ssniff Spezialdiäten GmbH |
| **Name** | Rat/Mouse – Maintenance extrudate | DIO – 60 kJ% fat (Lard) |
| **Catalogue number** | V1536 | E15742 |
| **Metabolizable energy [MJ/kg]** | **13.9** | **21.6** |
|  | | |
| **Metabolizable energy [kJ%]** | | |
| Fat | 10 | 60 |
| Protein | 23 | 20 |
| Carbohydrates | 67 | 20 |
|  | | |
| **Crude Nutrients [%]** | | |
| Crude fat | 3.6 | 34.6 |
| Crude protein | 19.1 | 24.4 |
| Crude fiber | 5.1 | 6.0 |
| Crude ash | 6.5 | 5.3 |
| Starch | 35.3 | 0.1 |
| Sugar | 4.8 | 9.4 |
| N free extracts | 55.9 | 26.3 |
|  | | |

**Supplementary Table 2** Gene names and sequences of the oligonucleotide primers

| **Name** | **Abbreviation** | **5´ sequence** | **3´ sequence** |
| --- | --- | --- | --- |
| Acetyl-CoA Carboxylase 1 | *Acc1* | AGCAGATCCGCAGCTTGGTCC | AGATGGGAGAGGCAGCCCGA |
| Actin Alpha 2, Smooth Muscle | *Acta2* | AACGAACGCTTCCGCTGCCC | GTGGTTTCGTGGATGCCCGCT |
| Adhesion G Protein-Coupled Receptor E1 | *Adgre1* | ACTGTGACCGGGGAGAAGAAGG | CAAGTTTGCCATCCGGTTACAGC |
| ATP Citrate Lyase | *Acly* | GTGGCGGGGAAGTGCTGTTTGA | TGTGCTCGGGCTGGGAAGGAC |
| Catalase | *Catalase* | CGAGCCCAGCCCTGACAAAATG | GAGCGCGGTAGGGACAGTTCACAG |
| Chemokine (C-C Motif) Ligand 2 | *Ccl2* | CATGCTTCTGGGCCTGCTGTT | CCTGCTGCTGGTGATCCTCTTGTA |
| Chemokine (C-C Motif) Ligand 3 | *Ccl3* | CAGCCGGAAGATTCCACGCC | GGACCCATGGAGGTTTGGGGG |
| CD68 Antigen | *Cd68* | CACTTCGGGCCATGTTTCTCTTG | AGGGGCTGGTAGGTTGATTGTCGT |
| Collagen Type I Alpha 1 Chain | *Col1a1* | GAAGGGGGCAAAGGTCCCCG | CCGGGAAGACCGACCACACC |
| Collagen Type III Alpha 1 Chain | *Col3a1* | ATGCCAGCCCCATGACTGTCC | AGGCCCGGCTGGAAAGAAGTC |
| Disintegrin and Metalloproteinase Domain-Containing Protein 8 | *Adam8* | GCTGCCAGGACCTCCGTGTA | GGAGGCTCGTAGACGCTGCT |
| Elongation factor 2  (**Liver housekeeping gene**) | *Ef2* | GAAACGCGCAGATGTCCAAAAGTC | GCCGGGCTGCAAGTCTAAGG |
| ELOVL family member 5, elongation of long chain fatty acids | *Elovl5* | CCTCTCGGGTGGCTGTTCTTCC | AGGCTTCGGCTCGGCTTGTC |
| Fatty Acid Synthase | *Fasn* | TGGGTGTGGAAGTTCGTCAG | GTCGTGTCAGTAGCCGAGTC |
| Glutathione peroxidase 1 | *Gpx1* | GGTTTCCCGTGCAATCAGTTCG | GAGCGCAGTGGGGTCGTCA |
| Glyceraldehyde-3-Phosphate Dehydrogenase  (**WAT housekeeping gene**) | *Gapdh* | CCCGGCATCGAAGGTGGAAGAGT | CTGACGTGCCGCCTGGAGAAAC |
| Interleukin 1 Beta | *Il1β* | TCCCCCACACGTTGACAGCTAGG | TCGGCCAAGACAGGTCGCTCA |
| Malic enzyme | *Me1* | TCTCACTGCCCAGGCTACACTAAC | CCTCCGTTAGCTTTGTTCTCTTTG |
| Microsomal glutathione S-transferase 1 | *Mgst1* | CCTGTTTGGCTGAGGAAGGGGA | TGCGCAGAGCCCACCTGAATGA |
| Nuclear factor (erythroid-derived 2)-like 2,= NFE2L2 | *Nrf2* | AAGGCTCCATCCTCCCGAACC | CTGAAAAGGCGGCTCAGCACC |
| Superoxid dismutase 2 | *Sod2* | GAAGCCCCTGTTTATCTGAGAGGCG | AACTGCACCATGAACTCACCGAGG |
| Thrombospondin 1 | *Thbs1* | CGAGCACCTGCGGAATGCAC | GCCGATGTGGCGAGGGTCAT |
| TIMP Metallopeptidase Inhibitor 1 | *Timp1* | TTTCCGTTCCTTAGGCGGCCC | GGGTTCCCCAGAAATCAACGAGACC |
| TIMP Metallopeptidase Inhibitor 2 | *Timp2* | AGAGAGCCAAACCGAGCCGTG | TGTGGTGAGGGGTGCTTGGC |
| TIMP Metallopeptidase Inhibitor 3 | *Timp3* | CCTGCCTCACATCAAGGTGCCA | CCTCCTCAACCCAAACAGCCGA |
| Tumor Necrosis Factor | *Tnfα* | AGCTGTCCCCACCTGGCCTCTC | CCCGTGGGGAGCAGAGGTTCAGT |

**Supplementary Table 3** List of annotated complex lipids and polar metabolites in liver samples

| Metabolite name (full) | Class |
| --- | --- |
| Carnitine; [M+H]+ | CAR |
| CAR 2:0; [M+H]+ | CAR |
| CAR 3:0; [M+H]+ | CAR |
| CAR 3:0-DC; [M+H]+ | CAR |
| CAR 4:0; [M+H]+ | CAR |
| CAR 4:0-OH; [M+H]+ | CAR |
| CAR 5:0; [M+H]+ | CAR |
| CAR 5:0-DC; [M+H]+ | CAR |
| CAR 5:0-M-DC; [M+H]+ | CAR |
| CAR 5:0-OH; [M+H]+ | CAR |
| CAR 6:0; [M+H]+ | CAR |
| CAR 6:0-DC; [M+H]+ | CAR |
| CAR 8:0; [M+H]+ | CAR |
| CAR 10:0; [M+H]+ | CAR |
| CAR 10:1; [M+H]+ | CAR |
| CAR 12:0; [M+H]+ | CAR |
| CAR 14:0; [M+H]+ | CAR |
| CAR 14:1; [M+H]+ | CAR |
| CAR 16:0; [M+H]+ | CAR |
| CAR 16:1; [M+H]+ | CAR |
| CAR 18:0; [M+H]+ | CAR |
| CAR 18:1; [M+H]+ | CAR |
| CAR 18:1-OH; [M+H]+ | CAR |
| CAR 18:2; [M+H]+ | CAR |
| CAR 20:0; [M+H]+ | CAR |
| CAR 20:1; [M+H]+ | CAR |
| CAR 20:4; [M+H]+ | CAR |
| CL 68:6; CL 16:1_18:2_16:1_18:2; [M-H]- | CL |
| CL 70:4; CL 16:0_18:1_18:1_18:2; [M-H]- | CL |
| CL 70:5; CL 16:0_18:2_18:1_18:2; [M-H]- | CL |
| CL 70:6; CL 16:1_18:2_18:1_18:2; [M-H]- | CL |
| CL 70:7; CL 16:1_18:2_18:2_18:2; [M-H]- | CL |
| CL 72:6; CL 18:1_18:2_18:1_18:2; [M-H]- | CL |
| CL 72:7; CL 18:1_18:2_18:2_18:2; [M-H]- | CL |
| CL 72:8; CL 18:2_18:2_18:2_18:2; [M-H]- | CL |
| CL 72:10; CL 33:1_39:9; [M-H]- | CL |
| CL 74:9; CL 18:2_18:2_18:2_20:3; [M-H]- | CL |
| CL 76:12; CL 36:4_40:8; [M-H]- | CL |
| Cer 34:1;2O; Cer 18:1;2O/16:0; [M+CH3COO]- | Cer |
| Cer 36:1;2O; Cer 18:1;2O/18:0; [M+CH3COO]- | Cer |
| Cer 38:1;2O; Cer 18:1;2O/20:0; [M+CH3COO]- | Cer |
| Cer 40:1;2O; Cer 18:1;2O/22:0; [M+CH3COO]- | Cer |
| Cer 40:2;2O; Cer 18:1;2O/22:1; [M+CH3COO]- | Cer |
| Cer 41:1;2O; Cer 18:1;2O/23:0; [M+CH3COO]- | Cer |
| Cer 42:1;2O; Cer 18:1;2O/24:0; [M+CH3COO]- | Cer |
| Cer 42:2;2O; Cer 18:1;2O/24:1; [M+CH3COO]- | Cer |
| Cer 42:3;2O; Cer 18:2;2O/24:1; [M+CH3COO]- | Cer |
| CoQ8; [M+H]+ | CoQ |
| CoQ9; [M+H]+ | CoQ |
| DG 32:1; DG 16:0_16:1; [M+NH4]+ | DG |
| DG 34:1; DG 16:0_18:1; [M+NH4]+ | DG |
| DG 34:2; DG 16:0_18:2; [M+NH4]+ | DG |
| DG 34:3; DG 16:1_18:2; [M+NH4]+ | DG |
| DG 35:2; DG 17:1_18:1; [M+NH4]+ | DG |
| DG 36:1; DG 18:0_18:1; [M+NH4]+ | DG |
| DG 36:2; DG 18:1_18:1; [M+NH4]+ | DG |
| DG 36:3; DG 18:1_18:2; [M+NH4]+ | DG |
| DG 36:4 (1); DG 18:2_18:2; [M+NH4]+ | DG |
| DG 36:4 (2); DG 16:0_20:4; [M+NH4]+ | DG |
| DG 38:2; DG 18:1_20:1; [M+NH4]+ | DG |
| DG 38:3 (1); DG 20:1_18:2; [M+NH4]+ | DG |
| DG 38:3 (2); DG 18:0_20:3; [M+NH4]+ | DG |
| DG 38:4 (1); DG 18:1_20:3; [M+NH4]+ | DG |
| DG 38:4 (2); DG 16:0_22:4; [M+NH4]+ | DG |
| DG 38:4 (3); DG 18:0_20:4; [M+NH4]+ | DG |
| DG 38:5 (1); DG 16:0_22:5; [M+NH4]+ | DG |
| DG 38:5 (2); DG 16:0_22:5; [M+NH4]+ | DG |
| DG 38:6; DG 16:0_22:6; [M+NH4]+ | DG |
| DG 38:7; DG 16:1_22:6; [M+NH4]+ | DG |
| DG 40:5; DG 18:1_22:4; [M+NH4]+ | DG |
| DG 40:6 (1); DG 18:1_22:5; [M+NH4]+ | DG |
| DG 40:6 (2); DG 18:1_22:5; [M+NH4]+ | DG |
| DG 40:6 (3); DG 18:0_22:6; [M+NH4]+ | DG |
| DG 40:7; DG 18:1_22:6; [M+NH4]+ | DG |
| DG 40:8; DG 18:2_22:6; [M+NH4]+ | DG |
| DGGA 38:6; DGGA 16:0_22:6; [M-H]- | DGGA |
| HexCer 40:2;2O; [M+CH3COO]- | HexCer |
| HexCer 34:1;2O; [M+CH3COO]- | HexCer |
| HexCer 38:1;2O; [M+CH3COO]- | HexCer |
| HexCer 40:1;2O; [M+CH3COO]- | HexCer |
| HexCer 41:1;2O; [M+CH3COO]- | HexCer |
| HexCer 42:1;2O; [M+CH3COO]- | HexCer |
| HexCer 42:2;2O; [M+CH3COO]- | HexCer |
| LPC 16:0; [M+H]+ | LPC |
| LPC 16:1; [M+H]+ | LPC |
| LPC 18:0; [M+H]+ | LPC |
| LPC 18:1; [M+H]+ | LPC |
| LPC 18:2; [M+H]+ | LPC |
| LPC 20:0; [M+H]+ | LPC |
| LPC 20:3; [M+H]+ | LPC |
| LPC 20:4; [M+H]+ | LPC |
| LPC 22:6; [M+H]+ | LPC |
| LPE 16:0; [M-H]- | LPE |
| LPE 18:0; [M-H]- | LPE |
| LPE 18:1; [M-H]- | LPE |
| LPE 20:4; [M-H]- | LPE |
| LPE 22:6; [M-H]- | LPE |
| LPG 18:1; [M-H]- | LPG |
| LPI 18:0; [M-H]- | LPI |
| LPI 20:4; [M-H]- | LPI |
| MG 18:2; [M+Na]+ | MG |
| PC 30:0; [M+H]+ | PC |
| PC 32:0; PC 16:0_16:0; [M+H]+ | PC |
| PC 32:1; PC 16:0_16:1; [M+H]+ | PC |
| PC 32:2; [M+H]+ | PC |
| PC 33:1; [M+H]+ | PC |
| PC 33:2; [M+H]+ | PC |
| PC 34:0; [M+H]+ | PC |
| PC 34:1; PC 16:0_18:1; [M+H]+ | PC |
| PC 34:2; PC 16:0_18:2; [M+H]+ | PC |
| PC 34:3 (1); PC 16:1_18:2; [M+H]+ | PC |
| PC 34:3 (2); [M+H]+ | PC |
| PC 34:4; [M+H]+ | PC |
| PC 35:1; [M+H]+ | PC |
| PC 35:2; [M+H]+ | PC |
| PC 35:3; [M+H]+ | PC |
| PC 35:4; [M+H]+ | PC |
| PC 36:1; PC 18:0_18:1; [M+H]+ | PC |
| PC 36:2; PC 18:0_18:2; [M+H]+ | PC |
| PC 36:3 (1); PC 18:1_18:2; [M+H]+ | PC |
| PC 36:3 (2); PC 16:0_20:3; [M+H]+ | PC |
| PC 36:3 (3); [M+H]+ | PC |
| PC 36:4 (1); PC 18:2_18:2; [M+H]+ | PC |
| PC 36:4 (2); PC 16:0_20:4; [M+H]+ | PC |
| PC 36:5 (1); [M+H]+ | PC |
| PC 36:5 (2); [M+H]+ | PC |
| PC 36:5 (3); [M+H]+ | PC |
| PC 36:6; [M+H]+ | PC |
| PC 37:2; [M+H]+ | PC |
| PC 37:4; PC 17:0_20:4; [M+H]+ | PC |
| PC 37:5; [M+H]+ | PC |
| PC 38:1; [M+H]+ | PC |
| PC 38:2; [M+H]+ | PC |
| PC 38:3 (1); PC 18:0_20:3; [M+H]+ | PC |
| PC 38:3 (2); [M+H]+ | PC |
| PC 38:4 (1); [M+H]+ | PC |
| PC 38:4 (2); PC 18:0_20:4; [M+H]+ | PC |
| PC 38:5 (1); PC 18:1_20:4; [M+H]+ | PC |
| PC 38:5 (2); [M+H]+ | PC |
| PC 38:6 (1); PC 18:2_20:4; [M+H]+ | PC |
| PC 38:6 (2); PC 16:0_22:6; [M+H]+ | PC |
| PC 38:7 (1); [M+H]+ | PC |
| PC 38:7 (2); [M+H]+ | PC |
| PC 39:4; [M+H]+ | PC |
| PC 40:3; [M+H]+ | PC |
| PC 40:4 (1); [M+H]+ | PC |
| PC 40:4 (2); [M+H]+ | PC |
| PC 40:5; [M+H]+ | PC |
| PC 40:6; PC 18:0_22:6; [M+H]+ | PC |
| PC 40:7 (1); [M+H]+ | PC |
| PC 40:7 (2); PC 18:1_22:6; [M+H]+ | PC |
| PC 40:8; PC 20:4_20:4; [M+H]+ | PC |
| PC 42:6; [M+H]+ | PC |
| PC 42:7; [M+H]+ | PC |
| PC 42:10; [M+H]+ | PC |
| PC O-32:0; [M+H]+ | EtherPC |
| PC O-34:1; [M+H]+ | EtherPC |
| PC O-36:4; [M+H]+ | EtherPC |
| PC O-38:5; [M+H]+ | EtherPC |
| PE 32:1; PE 16:0_16:1; [M-H]- | PE |
| PE 34:1; PE 16:0_18:1; [M-H]- | PE |
| PE 34:2; PE 16:0_18:2; [M-H]- | PE |
| PE 34:3 (1); PE 16:1_18:2; [M-H]- | PE |
| PE 34:3 (2); PE 16:1_18:2; [M-H]- | PE |
| PE 35:2; PE 17:0_18:2; [M-H]- | PE |
| PE 36:1; PE 18:0_18:1; [M-H]- | PE |
| PE 36:2; PE 18:0_18:2; [M-H]- | PE |
| PE 36:3; PE 18:1_18:2; [M-H]- | PE |
| PE 36:4 (1); PE 18:2_18:2; [M-H]- | PE |
| PE 36:4 (2); PE 16:0_20:4; [M-H]- | PE |
| PE 36:5 (1); PE 16:1_20:4; [M-H]- | PE |
| PE 36:5 (2); PE 16:0_20:5; [M-H]- | PE |
| PE 37:4; PE 17:0_20:4; [M-H]- | PE |
| PE 37:6; PE 15:0_22:6; [M-H]- | PE |
| PE 38:1; PE 18:0_20:1; [M-H]- | PE |
| PE 38:2 (1); PE 18:1_20:1; [M-H]- | PE |
| PE 38:2 (2); PE 20:0_18:2; [M-H]- | PE |
| PE 38:3 (1); PE 18:0_20:3; [M-H]- | PE |
| PE 38:3 (2); PE 18:0_20:3; [M-H]- | PE |
| PE 38:4 (1); PE 18:1_20:3; [M-H]- | PE |
| PE 38:4 (2); PE 18:0_20:4; [M-H]- | PE |
| PE 38:5 (1); PE 18:1_20:4; [M-H]- | PE |
| PE 38:5 (2); PE 16:0_22:5; [M-H]- | PE |
| PE 38:6 (1); PE 18:2_20:4; [M-H]- | PE |
| PE 38:6 (2); PE 16:0_22:6; [M-H]- | PE |
| PE 38:7 (1); PE 16:1_22:6; [M-H]- | PE |
| PE 38:7 (2); [M-H]- | PE |
| PE 39:4 (1); PE 19:0_20:4; [M-H]- | PE |
| PE 39:4 (2); PE 19:0_20:4; [M-H]- | PE |
| PE 39:6; PE 17:0_22:6; [M-H]- | PE |
| PE 40:4 (1); PE 18:0_22:4; [M-H]- | PE |
| PE 40:4 (2); PE 20:0_20:4; [M-H]- | PE |
| PE 40:5 (1); PE 20:1_20:4; [M-H]- | PE |
| PE 40:5 (2); PE 18:0_22:5; [M-H]- | PE |
| PE 40:6 (1); PE 18:1_22:5; [M-H]- | PE |
| PE 40:6 (2); PE 18:1_22:5; [M-H]- | PE |
| PE 40:6 (3); PE 18:0_22:6; [M-H]- | PE |
| PE 40:7; PE 18:1_22:6; [M-H]- | PE |
| PE 40:8; PE 18:2_22:6; [M-H]- | PE |
| PE 42:6; PE 20:0_22:6; [M-H]- | PE |
| PE 42:7; PE 20:1_22:6; [M-H]- | PE |
| PE O-36:2; PE O-18:1_18:1; [M-H]- | EtherPE |
| PE O-36:5; PE O-16:1_20:4; [M-H]- | EtherPE |
| PE O-38:4; PE O-18:0_20:4; [M-H]- | EtherPE |
| PE O-38:5 (1); PE O-16:1_22:4; [M-H]- | EtherPE |
| PE O-38:5 (2); PE O-18:1_20:4; [M-H]- | EtherPE |
| PE O-38:6 (1); PE O-18:2_20:4; [M-H]- | EtherPE |
| PE O-38:6 (2); PE O-16:1_22:5; [M-H]- | EtherPE |
| PE O-38:7; PE O-16:1_22:6; [M-H]- | EtherPE |
| PE O-40:5 (1); PE O-18:1_22:4; [M-H]- | EtherPE |
| PE O-40:5 (2); PE O-20:1_20:4; [M-H]- | EtherPE |
| PE O-40:6 (1); PE O-18:2_22:4; [M-H]- | EtherPE |
| PE O-40:6 (2); PE O-18:1_22:5; [M-H]- | EtherPE |
| PE O-40:7; PE O-18:1_22:6; [M-H]- | EtherPE |
| PE O-40:8; PE O-18:2_22:6; [M-H]- | EtherPE |
| PE O-42:7; PE O-20:1_22:6; [M-H]- | EtherPE |
| PE 40:7;O; PE 20:4_20:3;O; [M-H]- | OxPE |
| PE 40:8;2O; PE 20:5_20:3;2O; [M-H]- | OxPE |
| PE 42:8;2O; PE 22:5_20:3;2O; [M-H]- | OxPE |
| PE 44:7;O; PE 22:4_22:3;O; [M-H]- | OxPE |
| PE 44:8;2O; PE 22:5_22:3;2O; [M-H]- | OxPE |
| PG 32:1; PG 16:0_16:1; [M-H]- | PG |
| PG 34:1; PG 16:0_18:1; [M-H]- | PG |
| PG 34:2 (1); PG 16:1_18:1; [M-H]- | PG |
| PG 34:2 (2); PG 16:0_18:2; [M-H]- | PG |
| PG 34:3; PG 16:1_18:2; [M-H]- | PG |
| PG 36:2 (1); PG 18:1_18:1; [M-H]- | PG |
| PG 36:2 (2); PG 18:0_18:2; [M-H]- | PG |
| PG 36:3 (1); PG 18:1_18:2; [M-H]- | PG |
| PG 36:3 (2); PG 18:1_18:2; [M-H]- | PG |
| PG 36:4 (1); PG 18:2_18:2; [M-H]- | PG |
| PG 36:4 (2); PG 16:0_20:4; [M-H]- | PG |
| PG 38:4 (1); PG 18:1_20:3; [M-H]- | PG |
| PG 38:4 (2); PG 18:0_20:4; [M-H]- | PG |
| PG 38:5; PG 18:1_20:4; [M-H]- | PG |
| PG 38:6; PG 18:2_20:4; [M-H]- | PG |
| PG 38:7; PG 16:1_22:6; [M-H]- | PG |
| PG 40:5; PG 18:1_22:4; [M-H]- | PG |
| PG 40:6 (1); PG 18:1_22:5; [M-H]- | PG |
| PG 40:6 (2); PG 18:1_22:5; [M-H]- | PG |
| PG 40:7; PG 18:1_22:6; [M-H]- | PG |
| PG 40:8; PG 18:2_22:6; [M-H]- | PG |
| PG 42:8; PG 20:4_22:4; [M-H]- | PG |
| PG 42:9; PG 20:3_22:6; [M-H]- | PG |
| PG 42:10; PG 20:4_22:6; [M-H]- | PG |
| PG 44:10 (1); PG 22:4_22:6; [M-H]- | PG |
| PG 44:10 (2); PG 22:5_22:5; [M-H]- | PG |
| PG 44:11 (1); PG 22:5_22:6; [M-H]- | PG |
| PG 44:11 (2); PG 22:5_22:6; [M-H]- | PG |
| PG 44:12; PG 22:6_22:6; [M-H]- | PG |
| PI 34:2; PI 16:0_18:2; [M-H]- | PI |
| PI 34:3; PI 16:1_18:2; [M-H]- | PI |
| PI 36:1; PI 18:0_18:1; [M-H]- | PI |
| PI 36:2; PI 18:0_18:2; [M-H]- | PI |
| PI 36:3 (1); PI 16:0_20:3; [M-H]- | PI |
| PI 36:3 (2); PI 16:0_20:3; [M-H]- | PI |
| PI 36:4 (1); PI 18:2_18:2; [M-H]- | PI |
| PI 36:4 (2); PI 16:0_20:4; [M-H]- | PI |
| PI 36:5; PI 16:1_20:4; [M-H]- | PI |
| PI 37:4; PI 17:0_20:4; [M-H]- | PI |
| PI 38:3 (1); PI 18:0_20:3; [M-H]- | PI |
| PI 38:3 (2); PI 18:0_20:3; [M-H]- | PI |
| PI 38:4 (1); PI 18:1_20:3; [M-H]- | PI |
| PI 38:4 (2); PI 18:0_20:4; [M-H]- | PI |
| PI 38:5 (1); PI 18:1_20:4; [M-H]- | PI |
| PI 38:5 (2); [M-H]- | PI |
| PI 38:6 (1); PI 18:2_20:4; [M-H]- | PI |
| PI 38:6 (2); PI 16:0_22:6; [M-H]- | PI |
| PI 39:4; PI 19:0_20:4; [M-H]- | PI |
| PI 40:4 (1); PI 18:0_22:4; [M-H]- | PI |
| PI 40:4 (2); PI 20:0_20:4; [M-H]- | PI |
| PI 40:5 (1); PI 18:0_22:5; [M-H]- | PI |
| PI 40:5 (2); PI 18:0_22:5; [M-H]- | PI |
| PI 40:6; PI 18:0_22:6; [M-H]- | PI |
| PI 40:7; PI 18:1_22:6; [M-H]- | PI |
| PS 36:1; PS 18:0_18:1; [M-H]- | PS |
| PS 36:2; PS 18:0_18:2; [M-H]- | PS |
| PS 36:4; PS 16:0_20:4; [M-H]- | PS |
| PS 38:4; PS 18:0_20:4; [M-H]- | PS |
| PS 38:5; PS 18:1_20:4; [M-H]- | PS |
| PS 38:6; PS 16:0_22:6; [M-H]- | PS |
| PS 40:4; PS 18:0_22:4; [M-H]- | PS |
| PS 40:5; PS 18:0_22:5; [M-H]- | PS |
| PS 40:6; PS 18:0_22:6; [M-H]- | PS |
| PS 40:7; PS 18:1_22:6; [M-H]- | PS |
| SM 34:1;2O; [M+H]+ | SM |
| SM 36:1;2O; [M+H]+ | SM |
| SM 38:1;2O; [M+H]+ | SM |
| SM 38:4;2O; [M+H]+ | SM |
| SM 39:1;2O; [M+H]+ | SM |
| SM 40:1;2O; [M+H]+ | SM |
| SM 40:2;2O; [M+H]+ | SM |
| SM 41:1;2O; [M+H]+ | SM |
| SM 41:2;2O; [M+H]+ | SM |
| SM 42:1;2O; [M+H]+ | SM |
| SM 42:2;2O; [M+H]+ | SM |
| ST 27:1;O;S; [M-H]- | Cholesterol sulfate |
| Cholesterol; [M-H2O+H]+ | Cholesterol |
| TG 44:2; TG 10:0_16:0_18:2; [M+NH4]+ | TG |
| TG 46:0; TG 14:0_16:0_16:0; [M+NH4]+ | TG |
| TG 46:1; TG 14:0_16:0_16:1; [M+NH4]+ | TG |
| TG 46:2; TG 16:0_14:1_16:1; [M+NH4]+ | TG |
| TG 46:3; TG 12:0_16:1_18:2; [M+NH4]+ | TG |
| TG 48:0; TG 15:0_16:0_17:0; [M+NH4]+ | TG |
| TG 48:1; TG 14:0_16:0_18:1; [M+NH4]+ | TG |
| TG 48:2; TG 16:0_16:1_16:1; [M+NH4]+ | TG |
| TG 48:3; TG 14:0_16:1_18:2; [M+NH4]+ | TG |
| TG 48:4 (1); TG 14:1_16:1_18:2; [M+NH4]+ | TG |
| TG 48:4 (2); TG 16:0_16:1_16:3; [M+NH4]+ | TG |
| TG 49:1; TG 15:0_16:0_18:1; [M+NH4]+ | TG |
| TG 49:2 (1); TG 16:0_16:1_17:1; [M+NH4]+ | TG |
| TG 49:2 (2); TG 15:0_16:1_18:1; [M+NH4]+ | TG |
| TG 49:3; TG 15:0_16:1_18:2; [M+NH4]+ | TG |
| TG 50:0; TG 16:0_16:0_18:0; [M+NH4]+ | TG |
| TG 50:1; TG 16:0_16:0_18:1; [M+NH4]+ | TG |
| TG 50:2; TG 16:0_16:1_18:1; [M+NH4]+ | TG |
| TG 50:3; TG 16:0_16:1_18:2; [M+NH4]+ | TG |
| TG 50:4 (1); TG 16:1_16:1_18:2; [M+NH4]+ | TG |
| TG 50:4 (2); TG 16:0_18:1_16:3; [M+NH4]+ | TG |
| TG 50:5 (1); TG 16:1_16:2_18:2; [M+NH4]+ | TG |
| TG 50:5 (2); TG 16:0_18:2_16:3; [M+NH4]+ | TG |
| TG 51:1; TG 16:0_17:0_18:1; [M+NH4]+ | TG |
| TG 51:2; TG 16:0_17:1_18:1; [M+NH4]+ | TG |
| TG 51:3; TG 16:0_17:1_18:2; [M+NH4]+ | TG |
| TG 51:4; TG 16:1_17:1_18:2; [M+NH4]+ | TG |
| TG 52:1; TG 16:0_18:0_18:1; [M+NH4]+ | TG |
| TG 52:2; TG 16:0_18:1_18:1; [M+NH4]+ | TG |
| TG 52:3; TG 16:0_18:1_18:2; [M+NH4]+ | TG |
| TG 52:4; TG 16:1_18:1_18:2; [M+NH4]+ | TG |
| TG 52:5 (1); TG 16:1_18:2_18:2; [M+NH4]+ | TG |
| TG 52:5 (2); TG 16:0_16:1_20:4; [M+NH4]+ | TG |
| TG 52:6 (1); TG 16:0_18:2_18:4; [M+NH4]+ | TG |
| TG 52:6 (2); TG 14:0_16:0_22:6; [M+NH4]+ | TG |
| TG 52:7; TG 18:2_18:2_16:3; [M+NH4]+ | TG |
| TG 53:1; TG 16:0_19:0_18:1; [M+NH4]+ | TG |
| TG 53:2; TG 16:0_18:1_19:1; [M+NH4]+ | TG |
| TG 53:3; TG 17:0_18:1_18:2; [M+NH4]+ | TG |
| TG 53:4; TG 17:1_18:1_18:2; [M+NH4]+ | TG |
| TG 53:5; TG 18:1_17:2_18:2; [M+NH4]+ | TG |
| TG 54:1; TG 16:0_20:0_18:1; [M+NH4]+ | TG |
| TG 54:2; TG 16:0_18:1_20:1; [M+NH4]+ | TG |
| TG 54:3; TG 18:0_18:1_18:2; [M+NH4]+ | TG |
| TG 54:4; TG 16:0_18:1_20:3; [M+NH4]+ | TG |
| TG 54:5 (1); TG 16:0_18:2_20:3; [M+NH4]+ | TG |
| TG 54:5 (2); TG 16:0_18:1_20:4; [M+NH4]+ | TG |
| TG 54:6 (1); TG 18:1_18:2_18:3; [M+NH4]+ | TG |
| TG 54:6 (2); TG 16:0_18:2_20:4; [M+NH4]+ | TG |
| TG 54:6 (3); TG 16:0_16:0_22:6; [M+NH4]+ | TG |
| TG 54:7 (1); TG 16:1_18:2_20:4; [M+NH4]+ | TG |
| TG 54:7 (2); TG 16:0_16:1_22:6; [M+NH4]+ | TG |
| TG 54:8; TG 16:1_18:2_20:5; [M+NH4]+ | TG |
| TG 55:2; TG 16:0_18:1_21:1; [M+NH4]+ | TG |
| TG 55:3; TG 19:0_18:1_18:2; [M+NH4]+ | TG |
| TG 55:4; TG 16:0_18:1_21:3; [M+NH4]+ | TG |
| TG 55:5; TG 16:0_18:1_21:4; [M+NH4]+ | TG |
| TG 55:7; TG 16:0_17:1_22:6; [M+NH4]+ | TG |
| TG 56:1; TG 16:0_22:0_18:1; [M+NH4]+ | TG |
| TG 56:2; TG 16:0_18:1_22:1; [M+NH4]+ | TG |
| TG 56:3; TG 18:1_18:1_20:1; [M+NH4]+ | TG |
| TG 56:4; TG 18:1_20:1_18:2; [M+NH4]+ | TG |
| TG 56:5 (1); TG 16:0_18:1_22:4; [M+NH4]+ | TG |
| TG 56:5 (2); TG 18:0_18:1_20:4; [M+NH4]+ | TG |
| TG 56:6 (1); TG 16:0_18:1_22:5; [M+NH4]+ | TG |
| TG 56:6 (2); TG 16:0_18:1_22:5; [M+NH4]+ | TG |
| TG 56:7 (1); TG 16:0_18:2_22:5; [M+NH4]+ | TG |
| TG 56:7 (2); TG 16:0_18:1_22:6; [M+NH4]+ | TG |
| TG 56:8 (1); TG 18:2_18:2_20:4; [M+NH4]+ | TG |
| TG 56:8 (2); TG 16:0_18:2_22:6; [M+NH4]+ | TG |
| TG 56:9; TG 16:1_18:2_22:6; [M+NH4]+ | TG |
| TG 58:2; TG 16:0_18:1_24:1; [M+NH4]+ | TG |
| TG 58:3; TG 18:1_20:1_20:1; [M+NH4]+ | TG |
| TG 58:4; TG 20:0_18:1_20:3; [M+NH4]+ | TG |
| TG 58:5; TG 18:0_18:1_22:4; [M+NH4]+ | TG |
| TG 58:6; TG 16:0_18:1_24:5; [M+NH4]+ | TG |
| TG 58:7 (1); TG 16:0_18:1_24:6; [M+NH4]+ | TG |
| TG 58:7 (2); TG 18:0_18:1_22:6; [M+NH4]+ | TG |
| TG 58:8 (1); TG 18:1_18:2_22:5; [M+NH4]+ | TG |
| TG 58:8 (2); TG 16:0_18:2_24:6; [M+NH4]+ | TG |
| TG 58:9; TG 16:0_20:3_22:6; [M+NH4]+ | TG |
| TG 58:10 (1); TG 18:1_18:3_22:6; [M+NH4]+ | TG |
| TG 58:10 (2); TG 16:0_20:4_22:6; [M+NH4]+ | TG |
| TG 58:11; TG 16:0_20:5_22:6; [M+NH4]+ | TG |
| TG 60:3; TG 18:1_20:1_22:1; [M+NH4]+ | TG |
| TG 60:8; TG 18:1_18:1_24:6; [M+NH4]+ | TG |
| TG 60:10; TG 16:0_22:4_22:6; [M+NH4]+ | TG |
| TG 60:11; TG 16:0_22:5_22:6; [M+NH4]+ | TG |
| TG 60:12 (1); TG 20:4_20:4_20:4; [M+NH4]+ | TG |
| TG 60:12 (2); TG 16:0_22:6_22:6; [M+NH4]+ | TG |
| TG O-52:2; TG O-18:1_16:0_18:1; [M+NH4]+ | EtherTG |
| TG 52:3;1O; TG 16:0_18:1_18:2;1O; [M+NH4]+ | OxTG |
| 1-Methyladenosine; [M+H]+ | Polar |
| 1-Methylhistidine; [M+H]+ | Polar |
| 1-Methylnicotinamide; [M+H]+ | Polar |
| 2-Aminoadipic acid; [M+H]+ | Polar |
| 2-Hydroxybutyric acid; [M-H]- | Polar |
| 3'-Dephosphocoenzyme A; [M+H]+ | Polar |
| 3-Hydroxybutyric acid; [M-H]- | Polar |
| 3-Hydroxyisobutyric acid; [M-H]- | Polar |
| 3-Indoxylsulfate; [M-H]- | Polar |
| 3-Phenyllactic acid; [M-H]- | Polar |
| 3-Sulfopropanoic acid; [M-H]- | Polar |
| 4-Acetamidobutyric acid; [M-H]- | Polar |
| 4-Guanidinobutyric acid; [M+H]+ | Polar |
| 4-Hydroxyphenyllactic acid; [M-H]- | Polar |
| 4-Pyridoxic acid; [M+H]+ | Polar |
| 5-Aminovaleric acid betaine; [M+H]+ | Polar |
| 5-Methyltetrahydrofolic acid; [M-H]- | Polar |
| 5'-S-Methyl-5'-thioadenosine | Polar |
| Acetylcholine; [M]+ | Polar |
| Aconitic acid, cis-; [M-H]- | Polar |
| Adenosine 5'-diphosphoribose; [M+H]+ | Polar |
| Adenosine; [M+H]+ | Polar |
| Adenylosuccinic acid; [M-H]- | Polar |
| Ala-Gln; [M+H]+ | Polar |
| Ala-Ile; [M-H]- | Polar |
| Ala-Lys; [M+H]+ | Polar |
| Alanine; [M+H]+ | Polar |
| alpha-Hydroxyglutaric acid; [M-H]- | Polar |
| alpha-Ketoglutaric acid; [M-H]- | Polar |
| AMP (1); [M-H]- | Polar |
| AMP (2); [M-H]- | Polar |
| Arg-Ala; [M+H]+ | Polar |
| Arg-Gly; [M+H]+ | Polar |
| Arg-Leu; [M+H]+ | Polar |
| Asn-Leu; [M-H]- | Polar |
| Asparagine; [M+H]+ | Polar |
| Aspartic acid; [M+H]+ | Polar |
| Betaine; [M]+ | Polar |
| beta-Nicotinamide adenine dinucleotide, reduced; [M-H]- | Polar |
| beta-Nicotinamide adenine dinucleotide; [M+H]+ | Polar |
| Choline; [M]+ | Polar |
| Citric acid; [M-H]- | Polar |
| Citrulline; [M+H]+ | Polar |
| Creatine; [M+H]+ | Polar |
| Creatinine; [M+H]+ | Polar |
| Cytidine 5'-diphosphocholine; [M+H]+ | Polar |
| Cytidine 5'-monophosphate; [M-H]- | Polar |
| Cytidine; [M+H]+ | Polar |
| Dimethylarginine; [M+H]+ | Polar |
| Disaccharide (1); [M+Na]+ | Polar |
| Disaccharide (2); [M+Na]+ | Polar |
| Disaccharide (3); [M+Na]+ | Polar |
| Ergothioneine; [M+H]+ | Polar |
| Ethyl-beta-glucuronide; [M-H]- | Polar |
| Flavin adenine dinucleotide; [M+H]+ | Polar |
| Fumaric acid; [M-H]- | Polar |
| gamma-Butyrobetaine; [M]+ | Polar |
| gamma-Glu-Cys; [M+H]+ | Polar |
| Gln-Gly; [M+H]+ | Polar |
| Gln-Leu; [M-H]- | Polar |
| Glu-Ala; [M-H]- | Polar |
| Glucose 1-phosphate; [M+Na]+ | Polar |
| Glucose 6-phosphate; [M+Na]+ | Polar |
| Glucose; [M+Na]+ | Polar |
| Glu-Leu; [M-H]- | Polar |
| Glutamic acid; [M+H]+ | Polar |
| Glutamine; [M+H]+ | Polar |
| Glutaric acid; [M-H]- | Polar |
| Glutathione (oxidized); [M+H]+ | Polar |
| Glutathione (reduced); [M+H]+ | Polar |
| Glycero-3-phosphocholine; [M+H]+ | Polar |
| Glycerol 3-phosphate; [M-H]- | Polar |
| Glycine; [M+H]+ | Polar |
| Gly-Ile; [M-H]- | Polar |
| Gly-Leu; [M-H]- | Polar |
| Gly-Met; [M-H]- | Polar |
| Gly-Phe; [M-H]- | Polar |
| Guanidinosuccinic acid; [M+H]+ | Polar |
| Guanosine 5'-monophosphate; [M+H]+ | Polar |
| Guanosine; [M+NH4]+ | Polar |
| Hippuric acid; [M-H]- | Polar |
| His-Ser; [M+H]+ | Polar |
| Histamine; [M+H]+ | Polar |
| Histidine; [M+H]+ | Polar |
| Hypoxanthine; [M+H]+ | Polar |
| Ile-Gln; [M-H]- | Polar |
| Ile-Ile; [M-H]- | Polar |
| Ile-Lys; [M+H]+ | Polar |
| Ile-Phe; [M-H]- | Polar |
| Ile-Tyr; [M-H]- | Polar |
| Ile-Val; [M-H]- | Polar |
| Inosine 5'-monophosphate; [M+H]+ | Polar |
| Inosine; [M+H]+ | Polar |
| Isoleucine; [M+H]+ | Polar |
| Itaconic acid; [M-H]- | Polar |
| Lactic acid; [M-H]- | Polar |
| Lactobionic acid; [M-H]- | Polar |
| Leu-Ala; [M-H]- | Polar |
| Leucine; [M+H]+ | Polar |
| Leu-Glu; [M-H]- | Polar |
| Lysine; [M+H]+ | Polar |
| Malic acid; [M-H]- | Polar |
| Malonic acid; [M-H]- | Polar |
| Methionine; [M+H]+ | Polar |
| N,N-Dimethylglycine; [M+H]+ | Polar |
| N1-Acetylspermidine; [M+H]+ | Polar |
| N6,N6,N6-Trimethyllysine; [M+H]+ | Polar |
| N-Acetylalanine; [M-H]- | Polar |
| N-Acetylarginine; [M+H]+ | Polar |
| N-Acetylaspartic acid; [M-H]- | Polar |
| N-Acetylcitrulline; [M-H]- | Polar |
| N-Acetylglutamic acid; [M-H]- | Polar |
| N-Acetylglutamine; [M-H]- | Polar |
| N-Acetylhistamine; [M+H]+ | Polar |
| N-Acetylleucine; [M+H]+ | Polar |
| N-Acetyllysine; [M+H]+ | Polar |
| N-Acetylmethionine; [M-H]- | Polar |
| N-Acetylornithine; [M+H]+ | Polar |
| N-Acetylphenylalanine; [M-H]- | Polar |
| N-Acetyltryptophan; [M-H]- | Polar |
| N-Cinnamoylglycine; [M-H]- | Polar |
| N-epsilon-Dimethyllysine; [M+H]+ | Polar |
| N-epsilon-Methyllysine; [M+H]+ | Polar |
| N-gamma-Glutamylleucine; [M+H]+ | Polar |
| N-Glycolylneuraminic acid; [M-H]- | Polar |
| Nicotinamide riboside cation; [Cat]+ | Polar |
| Nicotinamide; [M+H]+ | Polar |
| N-Tigloylglycine; [M-H]- | Polar |
| Ophthalmic acid; [M+H]+ | Polar |
| Ornithine; [M+H]+ | Polar |
| Pantetheine; [M-H]- | Polar |
| Pantothenic acid; [M+H]+ | Polar |
| Phe-Gln; [M-H]- | Polar |
| Phenol sulfate; [M-H]- | Polar |
| Phenylalanine; [M+H]+ | Polar |
| Phe-Ser; [M-H]- | Polar |
| Phosphoenolpyruvic acid; [M-H]- | Polar |
| Pro-Ala; [M+H]+ | Polar |
| Proline; [M+H]+ | Polar |
| Putrescine; [M+H]+ | Polar |
| Pyridoxal 5'-phosphate; [M-H]- | Polar |
| Pyruvic acid; [M-H]- | Polar |
| Riboflavin; [M+CHO2]- | Polar |
| S-Adenosylhomocysteine; [M+H]+ | Polar |
| S-Adenosylmethionine; [M+H]+ | Polar |
| Serine; [M+H]+ | Polar |
| Ser-Leu; [M-H]- | Polar |
| Ser-Lys; [M-H]- | Polar |
| sn-glycerol-3-phosphoethanolamine; [M+Na]+ | Polar |
| Stachydrine (proline betaine); [M+H]+ | Polar |
| Sulfocholic acid; [M-H]- | Polar |
| Taurocholic acid (1); [M-H]- | Polar |
| Taurocholic acid (2); [M-H]- | Polar |
| Taurodeoxycholic acid; [M+H]+ | Polar |
| Taurohyocholic acid; [M+H]+ | Polar |
| Tetrasaccharide; [M+Na]+ | Polar |
| Thiamine; [M]+ | Polar |
| Threonic acid; [M-H]- | Polar |
| Threonine; [M+H]+ | Polar |
| Thr-Phe; [M-H]- | Polar |
| Thr-Tyr; [M-H]- | Polar |
| Thymidine 5'-monophosphate; [M-H]- | Polar |
| TMAO; [M+H]+ | Polar |
| Trigonelline; [M+H]+ | Polar |
| Trisaccharide; [M+Na]+ | Polar |
| Tryptophan; [M+H]+ | Polar |
| Tyr-Arg; [M+H]+ | Polar |
| Tyr-Gly; [M-H]- | Polar |
| Tyr-His; [M+H]+ | Polar |
| Tyrosine; [M+H]+ | Polar |
| UDP-Glc; [M-H]- | Polar |
| UDP-NAG; [M-H]- | Polar |
| Uracil; [M+H]+ | Polar |
| Urea; [M+H]+ | Polar |
| Uric acid; [M-H]- | Polar |
| Uridine 5'-diphosphoacetylglucosamine; [M+H]+ | Polar |
| Uridine 5'-monophosphate; [M+H]+ | Polar |
| Uridine; [M-H]- | Polar |
| Val-His; [M+H]+ | Polar |
| Valine; [M+H]+ | Polar |
| Val-Leu; [M-H]- | Polar |
| Val-Phe; [M-H]- | Polar |
| Val-Val; [M-H]- | Polar |
| Xanthine; [M+H]+ | Polar |
| Xanthosine; [M-H]- | Polar |

*Abbreviations*: CAR, acylcarnitine; CL, cardiolipin; Cer, ceramide; CoQ, coenzyme Q; DG, diacylglycerol; DGGA, diacylglyceryl glucuronide; HexCer, hexosylceramide; LPC, lysophophatidylcholine; LPE, lysophosphatidylethanolamine; LPG, lysophosphatidylglycerol; LPI, lysophosphatidylinositol; MG, monoacylglycerol; PC, phosphatidylcholine; EtherPC, ether-linked phosphatidylcholine; PE, phosphatidylethanolamine; EtherPE, ether-linked phosphatidylethanolamine; OxPE, oxidized phosphatidylethanolamine; PG, phosphatidylglycerol; PI, phosphatidylinositol; PS, phosphatidylserine; SM, sphingomyelin; TG, triacylglycerol; EtherTG, ether-linked triacylglycerol; OxTG, oxidized triacylglycerol.
